# Comparison of Drought and Heat Resistance Strategies among Six Populations of *Solanum chilense* and Two Cultivars of *Solanum lycopersicum*

**DOI:** 10.3390/plants10081720

**Published:** 2021-08-20

**Authors:** Rémi Blanchard-Gros, Servane Bigot, Juan-Pablo Martinez, Stanley Lutts, Gea Guerriero, Muriel Quinet

**Affiliations:** 1 Groupe de Recherche en Physiologie Végétale, Earth and Life Institute-Agronomy, Université Catholique de Louvain, 1348 Louvain-la-Neuve, Belgium; remi.blanchard@uclouvain.be (R.B.-G.); servane.bigot@uclouvain.be (S.B.); stanley.lutts@uclouvain.be (S.L.); 2 Instituto de Investigaciones Agropecuarias (INIA-La Cruz), La Cruz 2280454, Chile; jpmartinez@inia.cl; 3 Environmental Research and Innovation Department, Luxembourg Institute of Science and Technology, 4940 Hautcharage, Luxembourg; gea.guerriero@list.lu

**Keywords:** abiotic stress, plant physiology, stress-responsive genes, temperature increase, tomato, water stress, wild species

## Abstract

Within the tomato clade, *Solanum chilense* is considered one of the most promising sources of genes for tomato (*S. lycopersicum*) selection to biotic and abiotic stresses. In this study, we compared the effects of drought, high temperature, and their combination in two cultivars of *S. lycopersicum* and six populations of *S. chilense*, differing in their local habitat. Plants were grown at 21/19 °C or 28/26 °C under well-watered and water-stressed conditions. Plant growth, physiological responses, and expression of stress-responsive genes were investigated. Our results demonstrated strong variability among accessions. Differences in plant growth parameters were even higher among *S. chilense* populations than between species. The effects of water stress, high temperature, and their combination also differed according to the accession, suggesting differences in stress resistance between species and populations. Overall, water stress affected plants more negatively than temperature from a morpho-physiological point of view, while the expression of stress-responsive genes was more affected by temperature than by water stress. Accessions clustered in two groups regarding resistance to water stress and high temperature. The sensitive group included the *S. lycopersicum* cultivars and the *S. chilense* populations LA2931 and LA1930, and the resistant group included the *S. chilense* populations LA1958, LA2880, LA2765, and LA4107. Our results suggested that resistance traits were not particularly related to the environmental conditions in the natural habitat of the populations. The expression of stress-responsive genes was more stable in resistant accessions than in sensitive ones in response to water stress and high temperature. Altogether, our results suggest that water stress and high temperature resistance in *S. chilense* did not depend on single traits but on a combination of morphological, physiological, and genetic traits.

## 1. Introduction

Tomato (*Solanum lycopersicum* L.) is one of the most important vegetable crops in the world. Its production is estimated to be 180 million tons in 2019 [1], which makes it of major economic interest. However, like most cultivated plants, tomato is sensitive to abiotic stresses [2]. Global change predicts an increase in extreme climatic events and abiotic stresses such as heat and drought [3,4]. Among the abiotic stresses, heat and drought are the two main constraints to agricultural production that often co-occur in the field [4,5]. Co-occurrence of abiotic stresses leads to complex plant responses that cannot be deduced from single stresses [4,6,7]. Numerous studies have investigated the physiological and morphological impact of drought and heat applied individually on tomato, while the impact of combined stress has only recently been addressed [2,7,8,9,10]. Currently, the most relevant method to improve the tomato and make it more resistant to abiotic stresses (drought, high temperatures, salinity, etc.) is the introgression of resistant genes from wild relatives species [11,12]. The cultivated tomato is phylogenetically related to 13 wild tomato species native to South America that demonstrate considerable morphological and ecological diversity [13]. Compared with the large genetic variability found in wild tomato species, the genetic diversity of the cultivated tomato varieties is very limited due to their recent domestication from a small number of individuals [14,15]. Moreover, it was found that annual precipitations and temperature are significant factors in determining the distribution of wild tomato species [16].

Within the tomato clade, *Solanum chilense* is considered one of the most promising sources of genes for tomato selection to abiotic and biotic stresses due to its extremophile behavior and its high level of genetic variability [16,17,18,19]. *S. chilense* was also reported as one of the most polymorphic wild tomato species [20,21]. It grows naturally in northern Chile and southern Peru and originated from the Atacama Desert, one of the hottest and most arid deserts in the world [17,22]. This species exhibits a patchy population distribution across desert, high altitude, and coastal regions, and is divided into several populations that present resistance to more or less strong abiotic stresses and may be subject to large thermal amplitudes [23,24]. The *S. chilense* populations could be clustered in four groups, differing in their degree of genetic variation following the north-south colonization [24]. Genetic diversity is higher in the northern populations than in the southern coastal populations [21]. Moreover, evidence suggests that Pacific coastal populations and Andean inland populations represent separately evolving populations [21]. Signatures of positive selection in several abiotic stress-responsive genes have been identified at both the population and species levels [17]. In particular, annual precipitation explained more than 60% of *S. chilense* distribution [16]. This species has several adaptations for arid habitats including grayish pubescence and deep roots [20]. However, abiotic stress tolerance mechanisms remain largely unknown in this species compared to other wild tomato relatives [25,26]. Some studies investigated the physiological mechanisms underlying drought and salt tolerance in *S. chilense*, but they focused on one or a limited number of populations [23,25,27,28,29,30,31,32]. No study to date has investigated the resistance of this species to heat and the combination of drought and heat.

Abiotic stresses caused by environmental factors can affect the growth and development of crops with consequent decreases of crop yields and quality [33,34]. In cultivated tomato, abiotic stress causes about 70% yield loss, depending upon the plant stage and duration of the stress [35]. Plants respond to abiotic stresses through morphological, physiological, biochemical, and molecular changes [9,23,25]. Drought and heat stress are the most threatening abiotic stresses for crops and will be increasingly present in the context of global warming [36]. Drought is the direct result of reduced water supply and low soil moisture [37]. Cultivated tomato has a huge requirement of water [38]. Under drought, transpiration rate exceeds water uptake leading to cellular dehydration and osmotic stress [35]. Stomatal closure saves moisture in the plants but results in reduced photosynthesis and biomass production [9]. Overall, growth, development, and physiology of tomato plants are adversely affected by drought leading to a drastic reduction in yield [10,35]. Temperatures are expected to rise by 2–4.8 °C in the next few decades and the frequency of heat waves will increase [3]. Temperature increase could be beneficial to plants when it remains below the critical thresholds, but an increase above the temperature optimum is detrimental for plant growth and physiology [39]. With the increase of temperature, plants enhance their transpiration rate due to the water vapor gradient and need for cooling, this results in transpiration losses, stomata closure, and photosynthesis decrease [9,35]. In cultivated tomato, heat is responsible for a decrease in photosynthesis, flower wilting, low pollen viability, low fruit set, and a decrease in overall crop yield [40,41]. Heat is frequently associated with drought, which demonstrates the value of studying the combination of both stresses [10]. Indeed, plants tolerant to one stress may not necessarily be tolerant to the second stress or to their combination [7,9,10]. Often, the combination of drought and heat resulted in further injury, compared to a single stress in tomato [7,42]. Tomato exhibits both shared and unique responses to the combined drought and heat, as compared to single stresses [7,10,42].

In this study, the effects of drought, high temperature and their combination were compared in two cultivars of *S. lycopersicum* and six populations of *S. chilense*, belonging to different groups [24] and differing by their natural habitats. Plant growth, physiological and biochemical responses, and expression of drought- and heat-responsive genes were investigated. The aim was to identify the changes mediated by drought, high temperature, and their combination in *S. chilense*, and to compare them with *S. lycopersicum* in order to: (1) identify the most important traits that have been evolutionarily selected in these constrained environments, (2) identify the most resistant populations, and (3) investigate whether expression of drought and heat responsive genes are correlated with physiological resistance mechanisms. This study will not only help us understand the underlying physiological response mechanisms to combined stress in both species but will also provide valuable information for the breeding of abiotic stress-tolerant tomato.

## 2. Results

**Figure 1:**
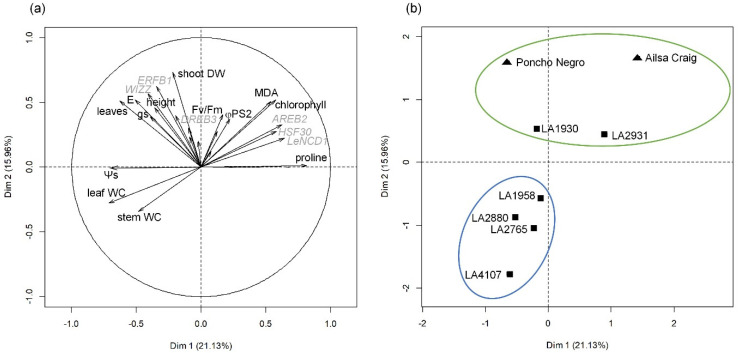
Principal component analysis (PCA) of growth parameters, physiological parameters, and gene expression of stress markers in *S. lycopersicum* and *S. chilense* subjected to two temperatures (21 °C vs. 28 °C) and water supply (well-watered vs. water-stressed) conditions. (**a**) Variable graph of PCA presenting growth and physiological parameters (black) and marker genes (grey); only significant parameters at *p* < 0.001 were included. (**b**) Individual graph presenting the accessions of *S. chilense* (square) and *S. lycopersicum* (triangle). Accessions were gathered in two groups. Dim 1 and Dim 2: dimension 1 and 2 of the PCA; DW: dry weight; gs: stomatal conductance; MDA: malondialdehyde; Ψs: osmotic potential; φPS2: efficiency of photosystem 2; WC: water content.

Plants of six populations of *S. chilense*, differing by their annual precipitations and temperature ranges (Table S1), and of two cultivars of *S. lycopersicum* (Ailsa Craig and Poncho Negro) were grown under four conditions in controlled chambers for 100 days. Plants were grown at either 21 °C under well-watered (21-WW) and water-stressed (21-WS) conditions or at 28 °C under well-watered (28-WW) and water-stressed (28-WS) conditions. A principal component analysis (PCA) was performed, in order to identify potential differences between accessions (Figure 1 and Figure S1). The PCA demonstrated that 21% of the variance was explained by axis 1 (Dim1) and 16% by axis 2 (Dim2). Axis 1 discriminates plants according to the treatments (Figure S1) and was mainly explained by, on one side, the leaf concentrations in proline, chlorophyll, and malondialdehyde (MDA) and the expression of genes *AREB2*, *HSF30*, and *LeNCED1*, which were found to be involved in response to water stress or heat [17,43] (Figure 1a). On the other side, axis 1 was explained by the osmotic potential (Ψs), the leaf and stem water content, the transpiration rate (E), the stomatal conductance (gs), and the number of leaves (Figure 1a). Axis 2 discriminates the accessions in two groups (Figure 1b) and was explained by the shoot dry weight, plant height, the leaf concentrations in MDA and chlorophyll, the transpiration rate, and the expression of the genes *ERFB1* and *WIZZ*, which are respectively involved in the response to temperature and water stress [43,44] (Figure 1a). The first group of accessions gathered the *S. lycopersicum* cv. Ailsa Craig and Poncho Negro along with the *S. chilense* populations LA1930 and LA2931 (Figure 1b). The second group consisted of the other *S. chilense* populations, namely LA1958, LA2880, LA2765, and LA4107 (Figure 1b). Results are presented according to these two groups in the following sections.

### 2.1. Plant Growth

**Figure 2:**
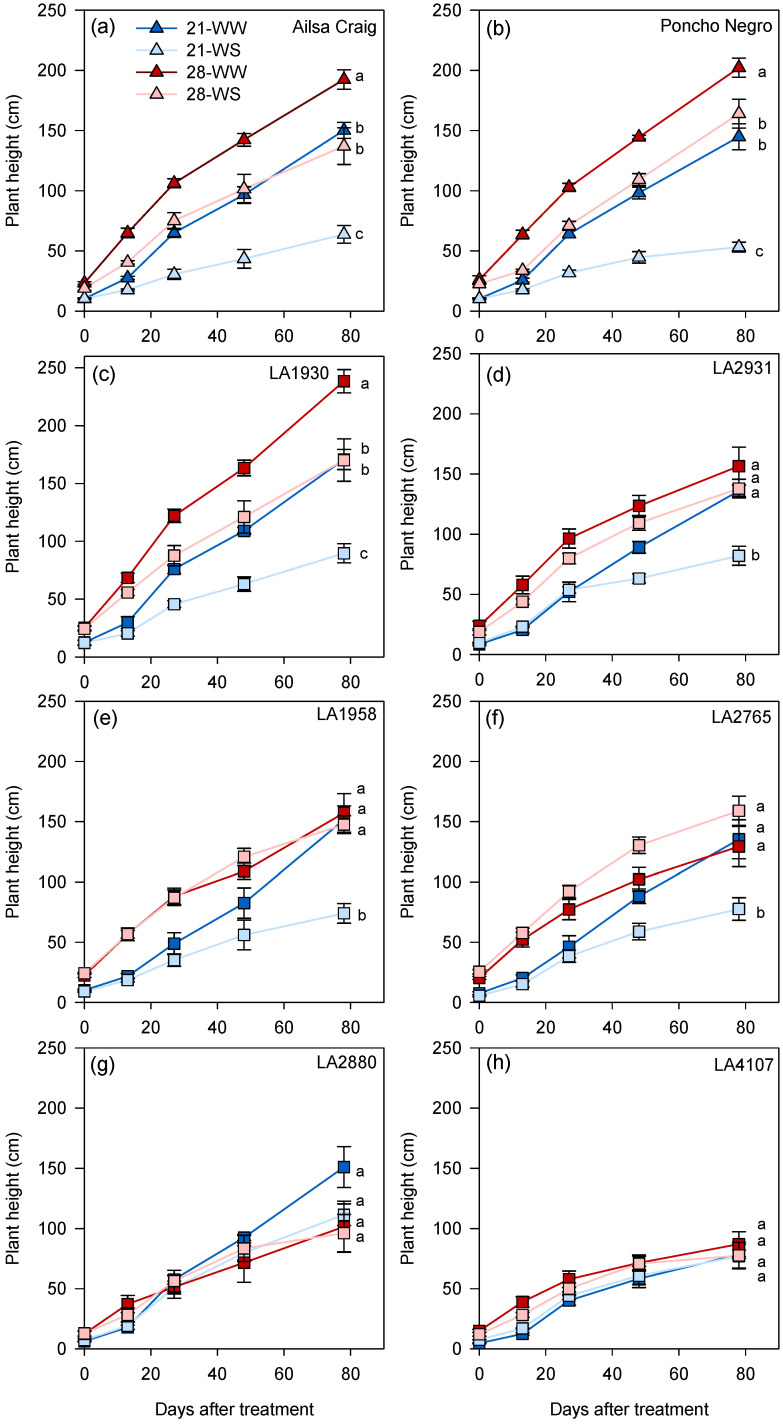
Effect of temperature and water stress on plant height of (**a**,**b**) *S. lycopersicum* cultivars and (**c**–**h**) *S. chilense* populations. Plants were grown at 21 °C under well-watered (21-WW) and water stress (21-WS) conditions or at 28 °C under well-watered (28-WW) and water-stressed (28-WS) conditions; triangle: *S. lycopersicum*, square: *S. chilense*. Treatments followed by different letters are significantly different at *p* < 0.05 for a same accession at the end of the experiment. Data ± SE.

**Figure 3:**
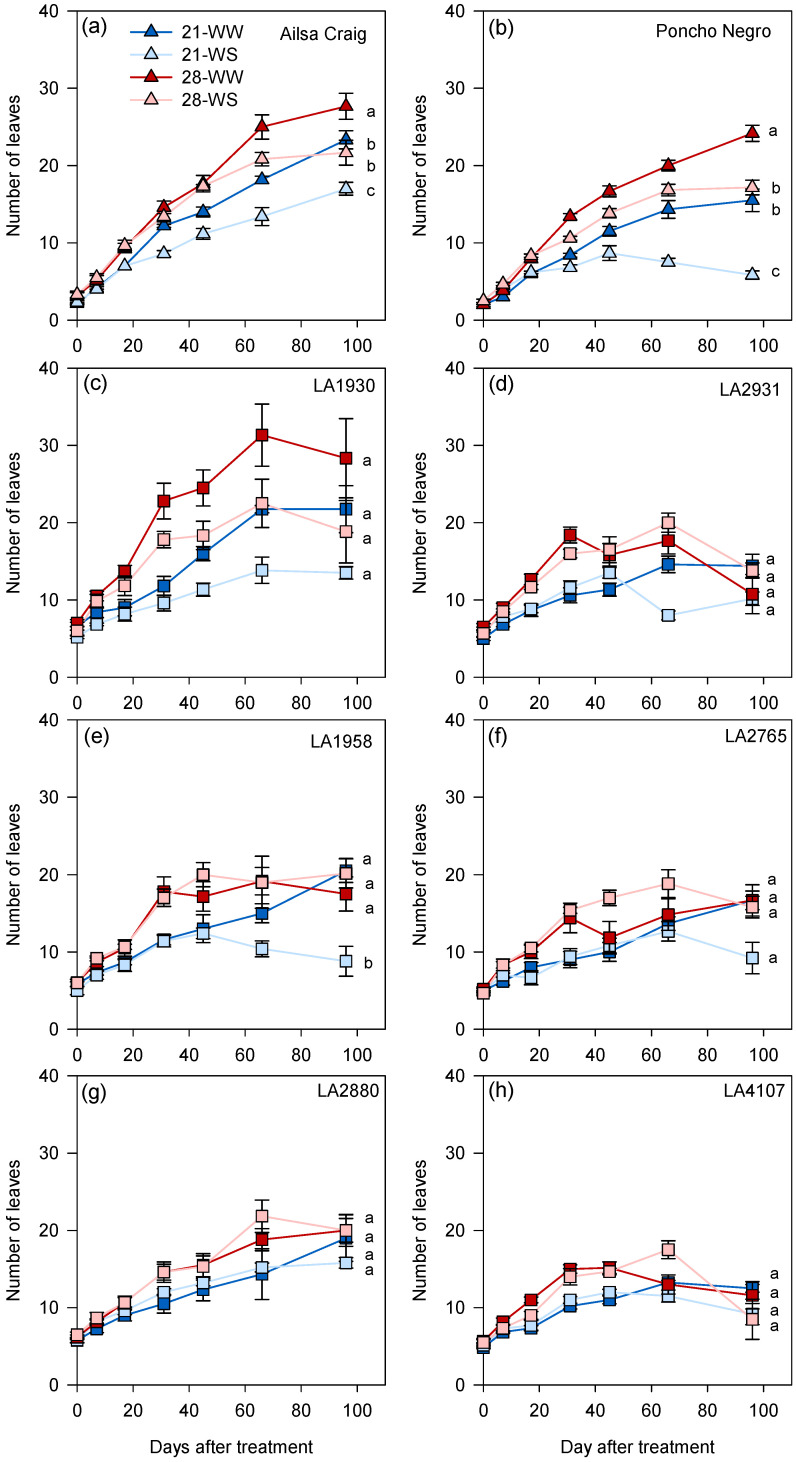
Effect of temperature and water stress on the leaf production of (**a**,**b**) *S. lycopersicum* cultivars and (**c**–**h**) *S. chilense* populations. Plants were grown at 21 °C under well-watered (21-WW) and water-stressed (21-WS) conditions or at 28 °C under well-watered (28-WW) and water-stressed (28-WS) conditions; triangle: *S. lycopersicum*, square: *S. chilense*. Treatments followed by different letters are significantly different at *p* < 0.05 for the same accession at the end of the experiment. Data ± SE.

Plant growth differed among accessions under control conditions (21-WW) and, overall, differences among *S. chilense* populations were higher than between *S. chilense* and *S. lycopersicum* (Table S2). Plant height was lower in *S. chilense* LA4107 compared to the other accessions (Figure 2 and Table S2). The mean leaf number on the main stem at the end of the experiment ranged from 12 in *S. chilense* LA4107 to 23 in *S. lycopersicum* cv. Ailsa Craig (Figure 3 and Table S2), while the number of ramifications on the main stem did not significantly vary among accessions (Table S2 and Table 1). The number of inflorescences on the main stem was higher in *S. lycopersicum* cv. Ailsa Craig compared to the other genotypes (Table S2 and Table 1). At the end of the experiment, the shoot dry weight ranged from less than 5 g in *S. chilense* LA2880 and LA4107 to more than 40 g in *S. chilense* LA1930 (Table S2).

The impact of temperature and water stress also differed according to the accession and the analyzed parameter (Table S3). Overall, temperature affected plant height and number of leaves, ramifications, and inflorescences on the main stem, while the water condition affected the number of leaves and ramifications on the main stem and the shoot dry weight (Table S3).

Regarding plant height, the temperature effect depended also on the water condition (Table S3). In *S. lycopersicum* cv. Ailsa Craig and Poncho Negro, and *S. chilense* LA1930, plants grown at 28-WW were taller than plants grown at 21-WW and 28-WS and the smallest plants were observed at 21-WS (Figure 2a,b,d). In *S. chilense* LA2931, LA1958, and LA2765, plants at 21-WS were smaller than in the other conditions (Figure 2c,e,f) while no differences were observed between treatments for *S. chilense* LA2880 and LA4107 (Figure 2g,h).

The number of leaves and ramifications on the main stem was more affected in *S. lycopersicum* than in *S. chilense* (Figure 3 and Table 1). In cv. Ailsa Craig and Poncho Negro, plants grown at 28-WW produced more leaves and ramifications while plants at 21-WS displayed the lowest number of leaves and ramifications (Figure 3a,b and Table 1). In *S. chilense*, the number of leaves was lower at 21-WS compared to the other conditions in LA1958 (Figure 3e) and the number of ramifications was higher at 28-WW compared to the other conditions in LA2880 (Table 1). No differences between treatments were observed for the other *S. chilense* populations (Figure 3c,d,f–h and Table 1).

Regarding the inflorescence production, water stress reduced the number of inflorescences on the main stem in *S. lycopersicum* cv. Ailsa Craig, while heat decreased it in *S. chilense* LA4107 (Table 1). The number of inflorescences was also reduced in 21-WS, 28-WW, and 28-WS compared to 21-WW in *S. chilense* LA1930, while no differences were observed among treatments for the other accessions (Table 1).

**Figure 4:**
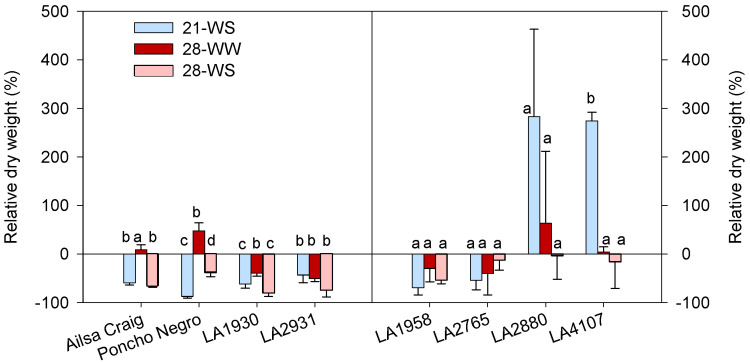
Effect of temperature and water stress on plant dry weight of *S. lycopersicum* cultivars and *S. chilense* populations: relative values to the control condition (21-WW) for each accession. Plants were grown at 21 °C under well-watered (21-WW) and water-stressed (21-WS) conditions or at 28 °C under well-watered (28-WW) and water-stressed (28-WS) conditions. Treatments followed by different letters are significantly different at *p* < 0.05 for the same accession (considering that the control plants are a). Data ± SE.

The effects of temperature and water condition on the shoot dry weight were both accession-dependent (Table S3). In *S. lycopersicum*, water stress decreased the shoot dry weight, while heat alone did not affect it (cv. Ailsa Craig) or even increased it (cv. Poncho Negro) (Figure 4). In *S. chilense*, both heat and water stress decreased the shoot dry weight in LA1930 and LA2931, while they did not affect the shoot dry weight in LA1958, LA2765, and LA2880. In *S. chilense* LA4107, the highest shoot dry weight was observed in 21-WS (Figure 4).

### 2.2. Plant Physiology

Regarding photosynthesis and water-related parameters under control conditions (Table S2), the chlorophyll concentration (CCI) was higher in the leaves of *S. lycopersicum* cv. Ailsa Craig than in *S. chilense* LA2765 at 21-WW, while there were no differences among accessions for the photosynthesis rate (A), the substomatal CO_2_ concentration (Ci), the transpiration rate (E), the stomatal conductance (gs), the water use efficiency (WUE), the efficiency of photosystem 2 (φPS2), the non-photochemical quenching (NPQ), and the osmotic potential (Ψs). Differences were observed among accessions for the leaf and stem water content (WC) at 21-WW. The leaf WC was higher in *S. chilense* LA4107 compared to LA2880, while the stem WC was lower in *S. chilense* LA1930 and LA2931 compared to the other accessions (Table S2). Regarding biochemical compounds at 21-WW, the concentration of proline was significantly higher in *S. chilense* LA1930 and significantly lower in *S. chilense* LA4107 and *S. lycopersicum* cv. Poncho Negro compared to the other accessions (Table S2). The malondialdehyde (MDA) concentrations, an indicator of lipid peroxidation, differed according to the accessions and ranged from 8.4 nmol/g FW in *S. chilense* LA4107 to 21.7 nmol/g FW in *S. lycopersicum* cv. Poncho Negro at 21-WW (Table S2).

Overall, the photosynthesis and water-related parameters were affected by temperature and water stress (Table S3): CCI, A, E, gs, leaf WC, and Ψs were affected by both temperature and water stress, while WUE and NPQ were only affected by temperature, stem WC was only affected by water stress, and Ci and φPS2 were neither affected by the temperature nor by the water stress. Moreover, a significant interaction between temperature and water stress was observed for A, Ci, gs, NPQ, leaf and stem WC, and Ψs (Table S3).

**Figure 5:**
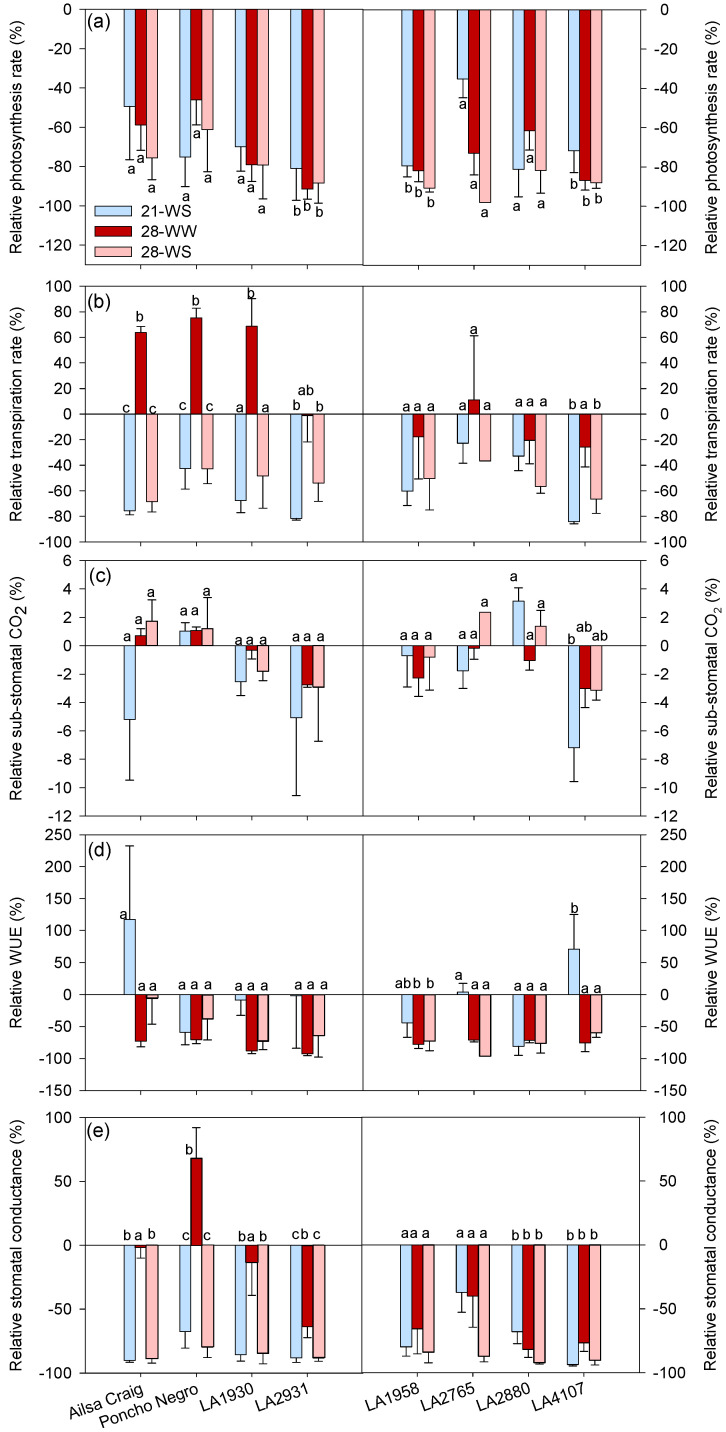
Effect of temperature and water stress on (**a**) photosynthesis rate (A), (**b**) transpiration rate (E), (**c**) substomatal CO_2_ concentration (Ci), (**d**) water use efficiency (WUE), and (**e**) stomatal conductance (gs) of *S. lycopersicum* cultivars and *S. chilense* populations: relative values to the control condition (21-WW) for each accession. Plants were grown at 21 °C under well-watered (21-WW) and water-stressed (21-WS) conditions or at 28 °C under well-watered (28-WW) and water-stressed (28-WS) conditions. Treatments followed by different letters are significantly different at *p* < 0.05 for the same accession (considering that the control plants are a). Data ± SE.

Comparison between accessions demonstrated that water stress, heat, and combined treatment significantly decreased A in *S. chilense* LA2931, LA1958, and LA4107 but did not significantly affect the other accessions (Figure 5a). However, Ci was only affected in *S. lycopersicum* cv. Ailsa Craig and in *S. chilense* LA4107 (Figure 5c). The effect of temperature and water stress on E and gs were accession dependent (Figure 5b and Table S3). In *S. lycopersicum*, E increased at 28-WW and decreased at 21-WS and 28-WS compared to 21-WW, while in *S. chilense*, heat increased E in LA1930 and water stress decreased E in LA2931 and LA4107 (Figure 5b). The gs also decreased with water stress in *S. lycopersicum* and it increased at 28-WW in cv. Poncho Negro (Figure 5e). In *S. chilense,* both heat and water stress decreased gs in LA2931, LA2880, and LA4107 (Figure 5e). The WUE was only significantly affected in *S. chilense* LA1958 and LA4107 (Figure 5d).

**Figure 6:**
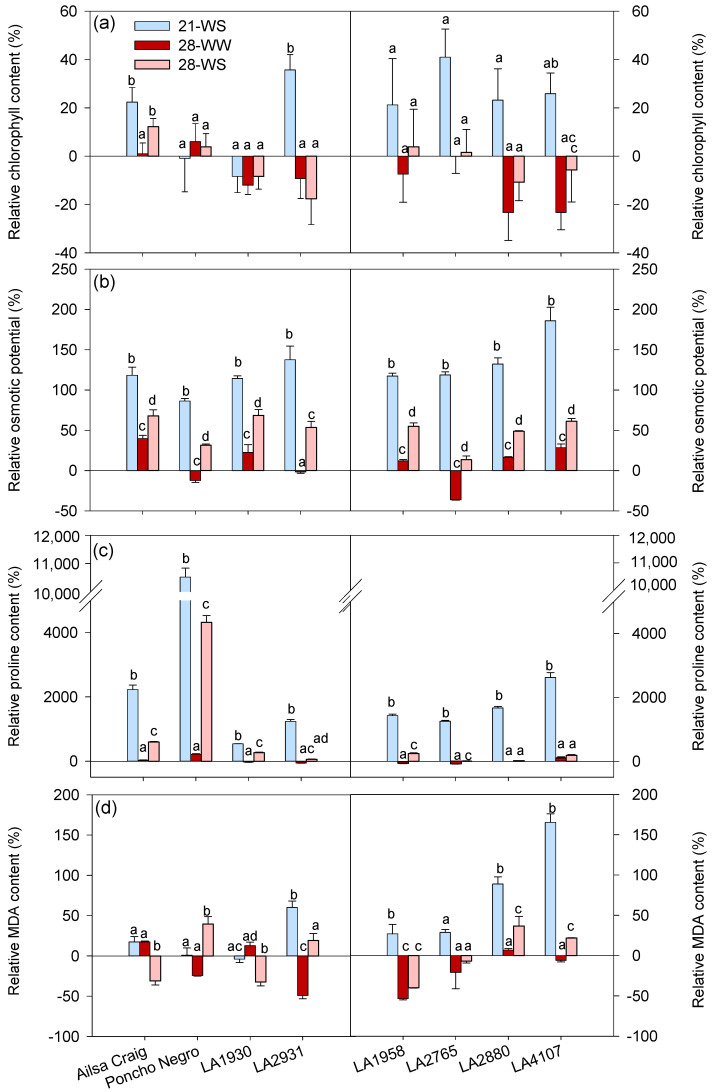
Effect of temperature and water stress on leaf (**a**) chlorophyll concentration, (**b**) osmotic pressure, (**c**) proline concentration, and (**d**) malondiadehyde concentration of *S. lycopersicum* cultivars and *S. chilense* populations: relative values to the control condition (21-WW) for each accession. Plants were grown at 21 °C under well-watered (21-WW) and water-stressed (21-WS) conditions or at 28 °C under well-watered (28-WW) and water-stressed (28-WS) conditions. Treatments followed by different letters are significantly different at *p* < 0.05 for the same accession (considering that the control plants are a). Data ± SE.

The chlorophyll content increased with water stress at 21 °C and 28 °C in *S. lycopersicum* cv. Ailsa Craig and at 21 °C in *S. chilense* LA2931, while heat decreased CCI in *S. chilense* LA4107; differences among treatments were not significant in the other accessions (Figure 6a). Regarding chlorophyll fluorescence parameters, φPS2 was not affected whatever the accession and NPQ decreased with heat in *S. chilense* LA2765 and in *S. lycopersicum* cv. Poncho Negro, but only under WS condition in the latter (Table 2).

In *S. lycopersicum*, water stress decreased the stem WC in cv. Ailsa Craig and heat decreased the stem WC in cv. Poncho Negro, while the leaf WC was not affected (Table 3). In *S. chilense*, water stress decreased the leaf WC of LA1930, LA2931, LA4107, and the stem WC of LA4107, but only at 21 °C (Table 3). 

The effects of temperature and water stress on the leaf Ψs, proline concentration, and MDA concentration were accession-dependent (Table S3, Figure 6b–d). Water stress significantly decreased Ψs in all accessions mainly at 21 °C, while heat decreased Ψs in *S. lycopersicum* cv. Ailsa Craig and *S. chilense* LA1930, LA1958, LA2880 and LA4107 and increased it in *S. lycopersicum* cv. Poncho Negro and *S. chilense* LA2765 (Figure 6b). The Ψs at 28-WS was thus intermediate between 21-WS and 28-WW in all accessions (Figure 6b). Consistent with the results of Ψs, proline concentration was significantly higher at 21-WS compared to 21-WW whatever the accession (Figure 6c). The proline concentration was similar at 28-WW and 21-WW in all accessions, and it was higher at 28-WS compared to 21-WW in *S. lycopersium* and *S. chilense* LA1930, LA1958, and LA2765 (Figure 6c). The effect of water stress was thus stronger at 21 °C than at 28 °C for Ψs and proline concentration, whatever the accession (Figure 6c,d). Moreover, the proline concentration was particularly increased by water stress in *S. lycopersicum* cv. Poncho Negro compared to the other accessions (Figure 6c). Regarding the MDA concentration, it increased with water stress at 21 °C in *S. chilense* LA2931, LA1958, LA2880, and LA4107 but not in *S. lycopersicum* (Figure 6d). Heat alone decreased MDA concentration in *S. chilense* LA2931 and LA1958, and combined treatment decreased it in *S. lycopersicum* cv. Ailsa Craig and *S. chilense* LA1930 and LA1958 but increased it in *S. lycopersicum* cv. Poncho Negro and *S. chilense* LA2880 and LA4107 compared to 21-WW (Figure 6d).

### 2.3. Expression of Water Stress and Heat Markers

**Figure 7:**
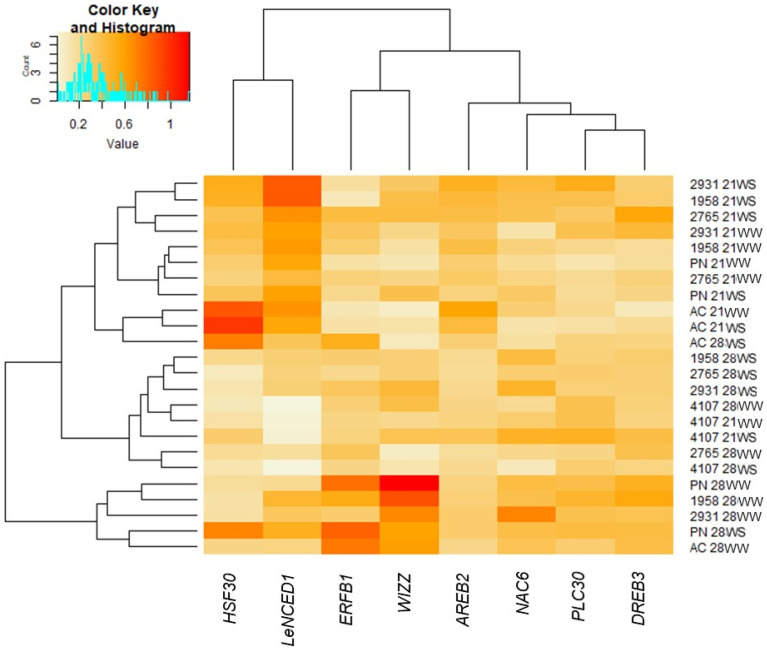
Effect of temperature and water stress on the expression of abiotic stress responsive genes in *S. lycopersicum* cultivars and *S. chilense* populations. Plants were grown at 21 °C under well-watered (21WW) and water-stressed (21WS) conditions or at 28 °C under well-watered (28 WW) and water-stressed (28WS) conditions. Means of 3 biological replicates, expression level relative to the reference genes (*PP2Acs: Solyc05g006590*, *CAC: Solyc08g006960*, and *TIP41: Solyc10g049850*) in log2 base. *S. lycopersicum* cv. Ailsa Craig (AC) and Poncho Negro (PN); *S. chilense* populations LA2931, LA1958, LA2765, and LA4107. *HSF30*: *Solyc08g062960*, *LeNCED1: Solyc07g056570*, *ERFB1: Solyc05g052040*, *WIZZ: Solyc03g116890*, *AREB2: Solyc11g044560*, *NAC6: Solyc10g055760*, *PLC30: Solyc04g082200*, and *DREB3: Solyc04g072900*.

The expression of 8 abiotic stress-responsive genes [17,43,44] was analyzed in the different *S. chilense* and *S. lycopersicum* accessions grown under 21-WW, 21-WS, 28-WW, and 28-WS conditions (Figure 7, Tables S4 and S5). *AREB2*, *DREB3*, *ERFB1*, *NAC6*, and *WIZZ* are regulatory genes and *HSF30*, *LeNCED1*, and *PLC30* are functional genes [17,43,44,45]. Signature of positive selection have been detected between *S. chilense* populations for some of these genes [17,45]. We observed differences of expression levels for these genes among accessions and treatments (Table S3).

Overall, the expression of *AREB2, DREB3, NAC6*, was mainly affected by temperature while the expression of *ERFB1*, *HSF30*, *LeNCED1*, and *WIZZ* was affected by both temperature and water stress (Table S3). A significant interaction between temperature and water stress was observed for the genes *DREB3*, *ERFB1*, *LeNCED1*, *NAC6*, *PLC30*, and *WIZZ* (Table S3). As observed on Figure 7, the genes were clustered in three groups according to the heat map. The first group included *AREB2*, *NAC6*, *PLC30*, and *DREB3*. The second group gathered *WIZZ* and *ERFB1* that displayed higher expression level at 28 °C in tomato cv. Ailsa Craig and Poncho Negro and in *S. chilense* LA 1958 and LA2931. The third group included *HSF30* that was more expressed in the tomato cv. Ailsa Craig at 21 °C and *LeNCED1* that was more expressed in the *S. chilense* populations LA2931 and LA1958 at 21 °C under water stress. The *PLC30* and *WIZZ* genes displayed, respectively, the lowest and the highest variability of expression levels among accessions and treatments.

The expression of all analyzed genes significantly differed among accessions (Figure 7 and Table S3). Regarding the accessions, Ailsa Craig displayed a significantly higher expression level of *AREB2* and *HSF30* and a significantly lower expression level of *DREB3*, compared to the other accessions. Poncho Negro displayed a significantly higher expression level of *WIZZ* and *HSF30*, compared to the other accessions. The gene *ERFB1* allowed a clear discrimination among the tomato cultivars and the *S. chilense* populations, with a higher expression level in *S. lycopersicum*. Clear discrimination among *S. chilense* populations was less obvious. The population LA4107 displayed, however, a significantly lower expression level of *LeNCED1* compared to all other accessions. The heat map divided the accessions and conditions into three main groups, mainly based on the temperature (Figure 7). The first group gathered mostly accessions grown at 21 °C. Inside this group, three subgroups could be isolated: (1) the *S. chilense* accessions LA2931 (21WW and 21WS), LA1958 (21WS), and LA2765 (21WS); (2) the *S. lycopersicum* cv. Poncho Negro (21WW and 21WS), and *S. chilense* populations LA1958 and LA2765 (21WW); (3) the *S. lycopersicum* cv. Ailsa Craig (21WW, 21WS, 28WS). The second group concerned mainly *S. chilense* accessions at 28 °C, including the populations LA4107 (21WW, 21WS, 28C, 28WS), LA1958 (28WS), LA2765 (28WS, 28WW), and LA2931 (28WS). The third group included the remaining accessions at 28 °C: the *S. lycopersicum* cvs. Poncho Negro (28WW and 28WS) and Ailsa Craig (28WW), and *S. chilense* populations LA1958 (28WW) and LA2931 (28WW). The *S. chilense* populations LA4107 and *S. lycopersicum* cv. Poncho Negro displayed, respectively, the lowest and highest gene expression variability among environmental conditions.

## 3. Discussion

We compared the resistance to water stress, high temperature and combined stress in several accessions of *S. lycopersicum* and *S. chilense*. Our results demonstrated strong variability among accessions even under control conditions (21-WW). At 21-WW, accessions mainly differed with respect to plant growth parameters, while photosynthesis and water status-related parameters were similar, with the exception of leaf and stem WC, chlorophyll concentration, and MDA concentration; these last three parameters were higher in *S. lycopersicum* than in *S. chilense*. Regarding plant growth parameters, differences among *S. chilense* populations were often higher than between species. *S. chilense* is indeed reported as one of the most polymorphic wild tomato species [20], and morphological differences were described depending on the location of the populations [21].

The effects of water stress, high temperature, and their combination also differed according to the accessions, suggesting differences in stress resistance and resistance mechanisms between species and populations. Overall, water stress affected plants more negatively than temperature increase and the effect of water stress was higher at 21 °C than at 28 °C. Zhou et al. [7,10,42] also reported a stronger impact of water stress compared to high temperature in cultivated tomato. Plant growth was boosted at 28 °C compared to 21 °C in most accessions in our experiment, mainly in *S. lycopersicum*. According to Sato et al. [46], optimal mean daily temperatures for tomato are between 21 °C and 24 °C, depending on their developmental stage [46]. Nankishore and Farrell [9] reported that cultivated tomato grows optimally between 20 °C and 30 °C, while, according to Ayenan et al. [39], optimal day and night temperatures for tomato development ranges from 21°C to 29.5°C and 18.5 °C to 21 °C, respectively. The applied temperature of 28/26 °C (day/night) was thus most probably not stressful for tomato, at least regarding the day temperature. Our results suggest that *S. lycopersicum* and *S. chilense* grow relatively well at 28 °C regarding vegetative growth, even though the mean temperature in the native habitat of the investigated *S. chilense* populations ranged from 12.3 °C to 19.6 °C. It should be mentioned, however, that some populations of *S. chilense* are subjected to large thermal amplitudes in the course of a day in their natural habitat [47]. *S. chilense* was reported to be cold tolerant [47] and Zhou et al. [48] demonstrated that it may acquire heat tolerance and survives some days above 50 °C. Regarding water stress, *S. chilense* was previously found to be a drought-resistant species [23], in agreement with the low precipitations observed in its natural habitat. Annual precipitation was suggested to explain more than 60% of *S. chilense* distribution [16]. However, *S. lycopersicum* has a high water requirement and is sensitive to water stress [35]. Our results also demonstrated that most *S. chilense* populations were more drought resistant than *S. lycopersicum*. In addition to *S. chilense*, other wild tomatoes display phenotypic and physiological adaptations to dry environments, although the adaptative traits may differ between species [16,23].

Accessions were divided in two groups according to the PCA. These two groups seem to differ regarding their stress resistance mainly regarding plant growth parameters. The *S. chilense* populations LA1930 and LA2931 were grouped with the *S. lycopersicum* cultivars, while the four other *S. chilense* populations were grouped together. Water stress and temperature affected the plant growth parameters more in the accessions of the first group than in the accessions of the second group, suggesting that the latter is more stress resistant than the former. Plant growth was boosted under water stress at 21 °C in LA4107. Moreover, the expression of drought and heat-responsive genes varied more with the environmental conditions in the sensitive accessions than in the resistant ones. However, the resistance to higher temperature and water stress seems unrelated to the environmental conditions of the natural habitat of the *S. chilense* populations. Indeed, the lowest precipitation level and the highest mean temperature are observed in the natural habitat of LA1930 which belonged to the sensitive group, and the natural habitat of the most resistant population LA4107 receives on average 3.5 times more precipitation and has a mean temperature 1.4 °C below the natural habitat of LA1930. It should be noted, however, that we have no information about underground water availability, which is likely key in determining the distribution of this species [21]. Moreover, we do not have a very precise description of the climate conditions of the different populations, but only average annual data, which does not allow us to make a real correlation between climatic conditions and morpho-physiological traits. Tapia et al. [23] did not observe correlations between drought physiological resistance traits and climate conditions in *S. chilense* and *S. peruvianum.* In the same vein, the chilling tolerance of *S. chilense* was not correlated with temperature in their native habitat [47]. However, climate variables, and particularly annual precipitation and annual temperature, were found to contribute to species distribution in the tomato clade [16]. This suggests that the environmental conditions explain more inter-species than intra-species distribution of wild tomato. Moreover, it was found that many taxa in the tomato clade are not monophyletic and that many individuals are admixed due to repeated hybridization [20]. In particular, discontinuous populations of *S. chilense* west of 72°, including LA1930, are most probably intermediate between *S. chilense* and *S. peruvianum* [20]. Böndel et al. [24] also found that the north western populations of *S. chilense* phylogenetically differed from the other populations. This could partly explain why LA1930 clustered apart from most *S. chilense* populations in our study. However, the population LA2931 that clustered with the sensitive group clearly belongs to the *S. chilense* species [24], and it grows in an intermediate environment regarding the environmental conditions compared to the other analyzed populations. Moreover, Raduski and Igic [21] reported that coastal Chilean populations are geographically, ecologically, morphologically, and genetically distinct from other populations, while LA4107, which belongs to this group, gathered with montane populations regarding abiotic stress resistance in our study. Phylogeny and environmental conditions do not therefore appear to be the main explanations for resistance traits in *S. chilense*.

We observed that the most resistant *S. chilense* populations were smaller and slow-growing compared to *S. lycopersicum* cultivars and sensitive *S. chilense* populations under control conditions. Their growth was also less affected, or even boosted, by water shortage and higher temperature. Plants of populations growing on Chilean coastal hills running along the Pacific Ocean, such as LA4107, are generally much shorter and decumbent than the montane populations of the Andean cordillera that are the densest with larger plants [21]. Moreover, they have unusually low prostrate growth, in contrast to the erect growth of the Andean inland populations [21]. However, plants of Peruvian populations west of 72°, including LA1930, are fast-growing and had significantly longer leaves, thicker stems and reduced lateral branching in comparison with most *S. chilense* populations [20]. These two populations were the most contrasting *S. chilense* populations regarding plant growth, drought, and higher temperature resistance in our study. Plants of LA1930 displayed a plant architecture more similar to *S. lycopersicum* than the other *S. chilense* populations, and shared common physiological strategies with *S. lycopersicum*, such as an increase of transpiration rate in response to higher temperature. Increasing transpiration is a strategy to enable the leaves to cool down under heat [49]. However, this strategy could have negative consequences in terms of water losses under drought conditions, and drought resistant plants have developed other strategies to enhance the ability for heat dissipation [50]. *S. chilense* has several adaptations for arid habitats including grayish pubescence and deep roots [20]. Martinez et al. [25] also reported that *S. chilense* is able to increase succulence while *S. lycopersicum* is unable to exhibit this adaptive feature. Succulence is an acclimation characteristic of plants to dry conditions [25]. Tapia et al. [23] found that *S. chilense* has a higher proline accumulation and stomatal rate (ratio of adaxial/abaxial stomatal density) than *S. lycopersicum* under drought conditions, and that proline significantly contributed to osmotic adjustment in this species under water stress [23]. We also observed an osmotic adjustment in response to water stress in our study and an increase in proline production, but the highest proline accumulation was observed in *S. lycopersicum* var. Poncho Negro. Most physiological parameters were affected by water stress and higher temperature in our study, but we did not clearly identify different physiological strategies between resistant and sensitive accessions. This corroborates the observation of Tapia et al. [23] that abiotic resistance in *S. chilense* was not associated with any single combination of traits. However, correlations between plant growth and physiological parameters differed between sensitive and resistant accessions (Figure S2). Plant growth parameters were positively correlated with the transpiration rate, the stomatal conductance, the osmotic potential, and the water content, and negatively correlated with proline concentration in sensitive accessions, while plant growth parameters were not clearly correlated with physiological parameters in resistant accessions. Moreover, proline and MDA concentrations were highly correlated in resistant accessions but not in sensitive ones. This could suggest that resistant accessions increased proline concentration in response to oxidative stress, proline acting as an antioxidant. Martinez et al. [25] reported that management of the oxidative status is a key mechanism allowing *S. chilense* (LA4107) to tolerate salinity. Similar mechanisms could be developed in resistant accessions to cope with water stress and higher temperature.

*S. chilense* is one of the tomato species with the greatest genetic variability and signatures of positive selection have been identified in several abiotic stress-responsive genes in this species [17,18]. Local adaptation to abiotic stresses was particularly apparent at the boundary of the species distribution in populations from coastal low-altitude and mountainous high-altitude regions [17,24]. However, the expression of these genes was not investigated in response to abiotic stress so far in *S. chilense*, to the best of our knowledge. In this study, we observed that the expression levels of stress-responsive genes were more affected by water stress and higher temperature in sensitive accessions than in resistant ones. A more stable expression of stress-responsive genes would suggest that the resistant accessions were not under strong stress in our conditions. Indeed, stress-responsive genes are activated in response to unfavorable conditions to allow plants to put in place specific responses to withstand the adverse conditions [51]. Although most of the investigated genes were described as drought-responsive genes [17,43], they were more affected by temperature than by water stress in our study, although *ERFB1*, *HSF30*, *LeNCED1*, and *WIZZ* were affected by both stresses. WRKY family proteins AP2/EREBP and AREB/ABF family transcription factors, heat shock factors (HSF), LEA proteins, and NAC genes are involved in the response of various environmental stresses [43,52,53]. *NAC6*, *WIZZ*, and *HSF30* were previously found to be overexpressed in drought-tolerant genotypes in a population of *S. pennellii* introgression lines [43]. *HSF30* was also upregulated after exposure to high-temperature stress in cultivated tomato [54]. *ERFB1* was previously found to be regulated by both heat stress and drought in cultivated tomato, although ERFs involved in response to temperature stress are usually different from those of other abiotic stresses in tomato [44]. Signatures of positive selection were reported for some of the genes investigated in this study [17]. According to Böndel et al. [17], *DREB3* was characterized by a high level of nucleotide diversity, suggesting a positive selection of this gene. On the contrary, the *AREB2* gene was characterized by rather low nucleotide diversity, suggesting that this gene is rather conserved in *S. chilense* [17]. However, the expression of both genes varied according to the accession and temperature in our conditions. This suggests that greater nucleotide diversity does not necessarily imply a greater difference in gene expression. Some populations also displayed higher signatures of positive selection than others [17,24]. In particular, Böndel et al. [17] observed consistent signatures of positive selection in several genes in the Chilean coastal populations including LA4107 [17]. This population displayed the lowest gene expression variability in our study and a different expression pattern compared to the other accessions, with a particular low expression of *LeNCD1*. Genetic differentiation was observed among *S. chilense* populations for this gene [45]. *LeNCED1* is involved in the abscisic acid pathway upstream of *PLC30*; this sequence also differed among *S. chilense* populations [17,45]. Both genes displayed very different patterns of nucleotide variation in *S. chilense* [45]. A positive selection was reported for *PLC30* in LA1958 and neighbor populations [17] and it was suggested that this gene was involved in adaptation to low temperature. We observed a higher expression of this gene at 28 °C compared to 21 °C in LA1958 and in other populations, suggesting that it could indeed be involved in response to temperature. Cold and heat stress response could indeed be correlated in some gene families [44].

Altogether, our study confirmed the high morpho-physiological variability among *S. chilense* populations. The investigated accessions gathered in two groups regarding resistance to water stress and higher temperature. The first group includes the *S. lycopersicum* cultivars and the *S. chilense* populations LA2931 and LA1930, which were more stress sensitive than the *S. chilense* populations LA1958, LA2880, LA2765, and LA4107, belonging to the second group. Resistance traits seemed not related to the environmental conditions in the natural habitat of the populations or to their phylogenetic similarity, but more to their growth habit. We did not clearly identify different physiological strategies between resistant and sensitive accessions, but correlations between plant growth and physiological parameters differed between both groups. Moreover, the expression of stress-responsive genes was more stable in resistant accessions than in sensitive ones in response to water stress and higher temperature. This suggests that water stress and high temperature resistance in *S. chilense* did not depend on single traits, but on a combination of morphological, physiological, and genetic traits.

## 4. Materials and Methods

### 4.1. Plant Culture and Growth Conditions

Seeds of the wild species *Solanum chilense* (TGRC accession number LA1930, LA1958, LA2765, LA2880, and LA2931, Table S1) and of the cultivated tomato (*Solanum lycopersicum*) cv. Ailsa Craig (TGRC accession number LA2838A) were obtained from the Tomato Genetics Resource Center (TGRC, University of California, Davis, CA, USA). Seeds of *S. lycopersicum* cv. Poncho Negro (accession number SLY 001, Chilean resistant cultivar) and *S. chilense* accession number LA4107 were kindly provided by INIA-La Cruz (La Cruz, Chile). The *S. chilense* populations were selected according to the four groups described by Böndel et al. [17] in order to compare populations differing in their natural habitat, and covering the whole range of the species. Seeds of *S. chilense* were pre-germinated for 96 h at 20 °C on Whatman paper moistened with sterilized water. The seeds were then germinated in peat compost (DCM; Amsterdam, The Netherlands) in a greenhouse under a photoperiod of 16/8h and day/night temperatures of 24/20 °C. Seeds of *S. lycopersicum* were germinated in peat compost under the same conditions one week after the sowing of *S. chilense* seeds in order that plantlets of both species were at the same developmental stage at the time of stress imposition. Twenty days after the sowing of *S. lycopersicum*, seedlings were transplanted into individual 7 cm × 7 cm pots in the same peat compost and placed in growth chambers at two temperature conditions (21/19 °C vs. 28/26 °C, day/night) and two water supply conditions (watering vs. water stress). Four different treatments were thus applied with 10 plants per accession and condition: 21/19 °C–well-watered (21-WW), 21/19 °C-water stress (21-WS), 28/26 °C–well-watered (28-WW), and 28/26 °C-water stress (28-WS) for 100 days. The light intensity was about 200 µmol·m^2^·s^−1^ from an HID lamp (HPI-T 400W Philips, Eindhoven, The Netherlands); the photoperiod was 16/8 h and the relative humidity was 80% ± 10%. The plants were transplanted a second time in 15 cm pots in the same peat compost two weeks later. Watering was applied to maintain a soil volumetric water content of 40% in control plants and 20% in water-stressed plants. Plants were regularly fertilized with a nutrient solution made of 15 g L^−1^ of a 16:18:21 N–P–K fertilizer. The soil water content was measured twice a week on 3 plants per accession and condition using a ProCheck sensor handheld reader (Decagon Devices; Pullman, DC, USA).

### 4.2. Plant Growth and Water Status Parameters

The height of the main stem, the number of leaves on the main stem, the number of lateral branches, and the number of inflorescences on the main stem were measured every two weeks along with the experiment on 5 plants per accession and condition. The growth of the aerial part was estimated at the end of the experiment, based on the fresh (FW) and dry (DW) weights of the stems and leaves of 3 individuals per accession and condition. The dry weight was determined after 72 h of incubation in an oven at 70 °C. The leaf and stem water content (WC) was calculated as (FW − DW)/FW × 100.

For the determination of osmotic potential (Ψs), leaves from 3 plants per accession and condition were collected, cut into small fragments, and placed in pierced Eppendorf tubes. The samples were then subjected to 3 freeze-thaw cycles; each tube was then inserted into a second Eppendorf tube and then centrifuged at 9000× *g* for 10 min at 4 °C. The osmolarity in the extracted sap was analyzed with an osmometer (Wescor vapro 5600 vapor pressure osmometer, Logan, UT, USA) and converted into Ψs according to the Van’t Hoff equation [55].

### 4.3. Photosynthesis Parameters

The chlorophyll fluorescence was measured on the upper side of the 5th mature leaf starting from the apex, on 5 plants per accession and condition using a fluorimeter (FMS II, Hansatech Instruments; Norfolf, UK). After an adaptation of at least 30 min in the dark, leaf portions were illuminated with a first pulse at 18,000 µmol m^−2^ s^−1^ followed by a constant intensity of actinic light of 660 µmol m^−2^ s^−1^ for 2 min; a new saturating flash of 18,000 µmol m^−2^ s^−1^ was subsequently applied. The efficiency of photosystem 2 (φPS2) and non-photochemical quenching (NPQ) were calculated according to Maxwell and Johnson [56].

Intercellular CO_2_ (Ci), stomatal conductance (gs), transpiration rate (E), and net photosynthesis rate (A) were measured by IRGA (InfraRed Gas Analyzer, LCI-SD 4-100, ADC BioScientific Limited; Hertfordshire, UK). The measurements were made on the 5th mature leaf starting from the apex, on 5 plants per accession and condition every 3 weeks. The instantaneous water use efficiency (WUE) was calculated as A/E.

The concentration of chlorophyll was measured using a chlorophyllometer (Opti-Sciences, CCM-200, Tyngsboro, MA, USA) on the 5th, 6th, and 7th mature leaves, starting from the apex, on 5 plants by accession and condition.

Physiological parameters were measured once a month and the presented results were obtained 2 months after stress imposition.

### 4.4. Malondialdehyde (MDA) and Proline Extraction and Quantification

For MDA extraction [57], 250 mg of frozen fresh leaves (4th–6th mature leaves, starting from the apex) were ground in liquid nitrogen to fine powder, mixed with 5 mL of trichloroacetic acid (5%), and centrifuged at 12,000× *g* for 10 min at 4 °C. The solution was filtered, then 2 mL of supernatant were removed and 2 mL of thiobarbituric acid (0.67%) were added. The samples were heated for 30 min in a water bath at 100 °C, cooled on ice, and centrifuged for 1 min at 12,000× *g*. The absorbance of the supernatant was measured at 532 nm using a spectrophotometer (Shimadzu; ‘s-Herlogenbosch, the Netherlands), and the non-specific absorbance due to turbidity was read at 600 nm and was subtracted from that read at 532 nm.

Proline was extracted and quantified as described by Bates et al. [58]: 200 mg of frozen fresh leaf material (4th–6th mature leaves, starting from the apex) previously ground in liquid nitrogen were extracted by adding 10 mL of 3% sulfosalicylic acid and incubation for 30 min at 70 °C. The solution was filtered, then 2 mL of the supernatant were removed, to which were added 2 mL of ninhydrin solution and 2 mL of glacial acetic acid. The samples were incubated for 1 h at 100 °C, and the reaction was stopped on ice. After adding 2 mL of toluene, the samples were strongly vortexed and the colored phase was read at 520 nm using a spectrophotometer (Shimadzu; ‘s-Herlogenbosch, The Netherlands).

Material for biochemical analysis was harvested 2 months after stress imposition.

### 4.5. Quantitative Real-Time PCR and Data Evaluation

The expression of 8 water stress and heat stress markers were analyzed. Reference and target genes were selected from *S. lycopersicum* according to the literature [17,43,44], and their coding sequences were searched on Phytozome and Sol genomics Network (SGN) websites.

The 5th mature leaf starting from the apex of 3 plants per accession and condition were pooled and ground in liquid nitrogen in triplicates, to obtain 3 biological replicates per accession and condition. Material for transcriptomic analysis was harvested 2 months after stress imposition. The RNA of the 72 samples was extracted from 100 mg of fresh material using the RNeasy Plant Mini Kit (Qiagen; Leusden, The Netherlands). An incubation step with DNase I (Qiagen, Leusden, The Netherlands) was carried out in order to eliminate the residual DNA. The concentration and purity of the RNA were measured using a NanoDrop ND-1000 spectrophotometer (Thermo scientific; Villebon-sur-Yvette, France). The RNA Integrity Number (RIN) was evaluated by electrophoresis with a 2100 Bioanalyzer (Agilent; Santa Clara, CA, USA) according to the manufacturer’s instructions, using the RNA 6000 nano chip (Agilent). One µg of RNA was then retrotranscribed into cDNA by reverse transcription using the Superscript II cDNA Synthesis Kit (Invitrogen; Carlsbad, CA, USA) according to the manufacturer’s instructions. Real time PCR were performed in triplicates for each biological replicate using 4 ng of cDNA, 5 µL of Takyon Low ROX SYBR Green (Eurogentec; Liege, Belgium), and 0.2 µM of primers listed in Table S5.

Reactions were prepared in 384 wells reaction (10 µL total volume). Plates were prepared using an automated dispensing device (epMotion 5073x, Eppendorf; Hambourg, Germany) with 3 technical replicates for each condition. A melt-curve analysis was performed to check the specific amplifications.

Primers were designed using Primer3Plus [59] and verified with the OligoAnalyzer tool from Integrated DNA Technologies (https://eu.idtdna.com/calc/analyzer, accessed on 1 June 2019) (Coralville, IA, USA). All the primers have a hairpin <60 °C, a self-dimer, and hetero-dimer with DeltaG < −7 kcal/mol. The pairs of primers were then aligned via ClustalOmega on all the other sequences, in order to verify that they did not match with other genes. After checking, 8 target genes (*AREB2*, *DREB3*, *ERFB1*, *HSF30*, *LeNCED1*, NAC6, pLC30-15, and *WIZZ*) and 5 reference genes (*PP2Acs*, *CAC*, *TIP41*, *UBI*, and *TUB*) were selected (Table S5). Normalization was carried out with *PP2Acs*, *CAC*, and *TIP41* which geNorm recognized as sufficient.

Primer efficiencies were determined by qPCR using serial five-fold dilutions (10, 2, 0.4, 0.08, 0.016, and 0.0032 ng/µL) of cDNA obtained from a pool of tissues from each conditions. R^2^ and amplification efficiencies (100% efficiency is defined as 2) were calculated using QuantStudio™ Design & Analysis Software v1.5.1 (Fisher Scientific; Merelbeke, Belgium).

### 4.6. Statistical Treatment

All statistical treatments were analyzed using RStudio [60] or SAS Enterprise Guide 8.3. Normality distributions and homoscedasticity were verified using Shapiro-Wilk and Levene’s test respectively, and data were transformed when required. A three-way analysis of variance (ANOVA) was performed to test the effect of the accession, temperature, and water conditions and their interactions on the analyzed parameters (plant number was mentioned as a repeated effect for the analysis of plant height and leaf number). Then, for each accession, data were analyzed using a two-way ANOVA with temperature and water conditions as main factors, and interaction between both factors. Post-hoc comparisons between treatments for a same accession were performed using a Tukey test. Differences between accessions and conditions were also visualized using principal component analysis (PCA) using the ‘FactoMineR’ package. Heat maps were performed using the ‘gplots’ package to visualize the gene expression results. Relative differences between treatments were calculated as (Vs − Vc)/Vc × 100, where Vc is the value for the 21-WW plants and vs. is the value for stressed plants (either 21-WS, 28-WW, or 28-WS). Data were presented as means ± standard errors.

## Figures and Tables

**Table 1:** Effect of temperature and water stress on the number of ramifications and inflorescences of *S. lycopersicum* cultivars and *S. chilense* populations. For a given accession, treatments followed by different letters are significantly different at *p* < 0.05. Data ± SE.

Accession	21-WW	21-WS	28-WW	28-WS
Number of ramifications				
Ailsa Craig	4.5 ± 0.3 ab	3.0 ± 0.5 b	5.2 ± 0.6 a	3.3 ± 0.3 b
Poncho Negro	3.2 ± 0.5 ab	0.8 ± 0.2 c	4.0 ± 0.5 a	2.5 ± 0.2 b
LA1930	4.8 ± 2.8 ab	0.8 ± 0.6 b	11.2 ± 3.0 a	5.3 ± 3.0 ab
LA2931	8.2 ± 1.1 a	9.2 ± 0.7 b	15.5 ± 3.0 b	11.2 ± 2.4 b
LA1958	8.3 ± 2.9 a	5.0 ± 1.6 a	7.7 ± 1.2 a	9.5 ± 1.8 a
LA2765	8.0 ± 2.9 a	9.8 ± 1.8 a	7.3 ± 2.0 a	9.0 ± 1.2 a
LA2880	5.7 ± 1.8 b	4.4 ± 1.0 b	11.5 ± 2.0 a	6.3 ± 0.8 b
LA4107	9.7 ± 2.7 a	10.8 ± 1.2 a	10.8 ± 1.6 a	8.6 ± 1.3 a
Number of inflorescences on the main stem				
Ailsa Craig	5.50 ± 0.34 a	3.00 ± 0.45 b	4.83 ± 0.17 a	3.33 ± 0.33 b
Poncho Negro	2.50 ± 0.67 a	1.33 ± 0.49 a	2.67 ± 0.33 a	1.67 ± 0.42 a
LA1930	0.80 ± 0.37 a	0.00 ± 0.00 b	0.00 ± 0.00 b	0.17 ± 0.17 b
LA2931	1.40 ± 0.75 a	3.00 ± 0.58 a	2.67 ± 0.88 a	2.83 ± 1.01 a
LA1958	1.80 ± 0.92 a	1.00 ± 0.55 a	0.17 ± 0.17 a	1.83 ± 0.60 a
LA2765	2.50 ± 0.50 a	2.33 ± 0.67 a	1.50 ± 0.96 a	1.37 ± 0.56 a
LA2880	1.75 ± 0.75 a	2.60 ± 0.75 a	0.20 ± 0.20 a	0.83 ± 0.65 a
LA4107	2.67 ± 0.95 ab	3.33 ± 0.49 a	1.00 ± 0.45 b	0.50 ± 0.50 b

**Table 2:** Effect of temperature and water stress on chlorophyll fluorescence parameters of *S. lycopersicum* cultivars and *S. chilense* populations. For a given accession, treatments followed by different letters are significantly different at *p* < 0.05. Data ± SE.

Accession	21-WW	21-WS	28-WW	28-WS
Efficiency of photosystem 2 (φPS2)				
Ailsa Craig	0.78 ± 0.02 a	0.82 ± 0 a	0.81 ± 0.01 a	0.80 ± 0.01 a
Poncho Negro	0.61 ± 0.14 a	0.59 ± 0.11 a	0.76 ± 0.02 a	0.79 ± 0.01 a
LA1930	0.81 ± 0.01 a	0.76 ± 0.05 a	0.81 ± 0.01 a	0.75 ± 0.05 a
LA2931	0.73 ± 0.04 a	0.83 ± 0.01 a	0.74 ± 0.04 a	0.77 ± 0.02 a
LA1958	0.75 ± 0.04 a	0.78 ± 0.05 a	0.77 ± 0.04 a	0.70 ± 0.08 a
LA2765	0.71 ± 0.09 a	0.74 ± 0.08 a	0.72 ± 0.07 a	0.77 ± 0.02 a
LA2880	0.57 ± 0.10 a	0.52 ± 0.13 a	0.73 ± 0.02 a	0.79 ± 0.02 a
LA4107	0.74 ± 0.06 a	0.84 ± 0.01 a	0.74 ± 0.04 a	0.73 ± 0.03 a
Non-photochemical quenching (NPQ)				
Ailsa Craig	0.32 ± 0.09 a	0.23 ± 0.02 a	0.23 ± 0.01 a	0.19 ± 0.06 a
Poncho Negro	0.30 ± 0.03 ab	0.47 ± 0.08 a	0.28 ± 0.05 ab	0.20 ± 0.02 b
LA1930	0.25 ± 0.05 a	0.23 ± 0.02 a	0.26 ± 0.03 a	0.30 ± 0.06 a
LA2931	0.27 ± 0.05 a	0.25 ± 0.01 a	0.43 ± 0.17 a	0.16 ± 0.02 a
LA1958	0.27 ± 0.04 a	0.30 ± 0.03 a	0.32 ± 0.06 a	0.20 ± 0.03 a
LA2765	0.24 ± 0.04 a	0.37 ± 0.07 a	0.18 ± 0.04 b	0.16 ± 0.04 b
LA2880	0.34 ± 0.02 a	0.32 ± 0.10 a	0.32 ± 0.10 a	0.20 ± 0.03 a
LA4107	0.27 ± 0.05 a	0.20 ± 0.03 a	0.24 ± 0.05 a	0.17 ± 0.06 a

**Table 3:** Effect of temperature and water stress on water content of *S. lycopersicum* cultivars and *S. chilense* populations. For a given accession, treatments followed by different letters are significantly different at *p* < 0.05. Data ± SE.

Accession	21-WW	21-WS	28-WW	28-WS
Leaf water content (%)				
Ailsa Craig	90.30 ± 0.71 a	83.56 ± 2.54 a	89.22 ± 0.28 a	83.86 ± 1.39 a
Poncho Negro	89.88 ± 0.98 a	83.13 ± 2.92 a	89.82 ± 1.31 a	81.15 ± 2.43 a
LA1930	88.88 ± 0.55 a	82.61 ± 0.94 b	91.35 ± 0.40 a	91.79 ± 1.37 a
LA2931	89.06 ± 0.84 a	77.54 ± 2.52 b	89.61 ± 0.21 a	90.82 ± 1.77 a
LA1958	90.70 ± 0.95 a	88.31 ± 3.94 a	90.50 ± 0.47 a	91.46 ± 0.99 a
LA2765	90.02 ± 1.50 a	83.87 ± 1.01 a	90.11 ± 0.78 a	86.46 ± 2.34 a
LA2880	87.12 ± 1.61 a	85.47 ± 4.08 a	93.18 ± 2.95 a	91.97 ± 1.52 a
LA4107	93.11 ± 0.87 a	82.16 ± 0.35 b	91.10 ± 0.20 a	92.37 ± 2.22 a
Stem water content (%)				
Ailsa Craig	92.06 ± 0.32 a	86.97 ± 0.10 b	89.03 ± 0.30 a	87.23 ± 1.32 b
Poncho Negro	91.94 ± 0.61 a	90.12 ± 0.73 a	86.99 ± 0.52 b	85.45 ± 1.56 b
LA1930	85.80 ± 1.12 a	86.37 ± 1.12 a	89.54 ± 0.64 a	89.97 ± 2.02 a
LA2931	86.60 ± 0.80 a	67.01 ± 15.36 a	78.60 ± 12.35 a	90.47 ± 2.32 a
LA1958	90.35 ± 1.34 a	89.83 ± 3.23 a	90.58 ± 0.78 a	88.76 ± 0.19 a
LA2765	91.58 ± 2.09 a	2.81 ± 1.62 a	90.44 ± 1.61 a	87.21 ± 1.73 a
LA2880	92.48 ± 2.35 a	86.33 ± 4.65 a	92.76 ± 3.46 a	91.90 ± 2.22 a
LA4107	93.42 ± 1.112 a	83.54 ± 0.35 b	94.02 ± 2.06 a	91.96 ± 1.92 a

## Data Availability

The data presented in this study are available in the article and supplemental data.

## Supplementary Materials

The following are available online at https://www.mdpi.com/article/10.3390/plants10081720/s1: Table S1. Origin of the *S. chilense* populations, and Figure S1. Principal component analysis (PCA) of growth parameters, physiological parameters, and gene expression of stress markers in *S. lycopersicum* and *S. chilense* subjected to two temperatures (21 °C vs. 28) and water supply (well-watered vs. water- stressed) conditions. (A) Variable graph of PCA displaying growth and physiological parameters (black) and marker genes (grey); only significant parameters at *p* < 0.001 were included. (B,D) Individual graphs displaying the accessions of *S. chilense* (square) and *S. lycopersicum* (triangle). (C,E) Individual graphs displaying the treatments. Only mean individuals are presented in (B,C) and all individuals are presented in (D,E); Table S2. Plant growth and physiological parameters of *S. lycopersicum* (cv. Ailsa Craig and Poncho Negro) and *S. chilense* (populations LA1930, LA2931, LA1958, LA2765, LA2880, LA4107) grown under control conditions (21 °C/19 °C, 40% soil VWC); Table S3. Results of statistical analysis (ANOVA3) for the analyzed parameters; Table S4. Heat and drought effect on the expression of abiotic stress responsive genes in *S. lycopersicum* cultivars and *S. chilense* populations; Figure S2. Correlation plots between plant growth and physiological parameters in (A) sensitive accessions and (B) resistant accessions. Only significant Pearson correlations at 5% were included; and Table S5. List of genes and primers used in this study.
