# Supplementary material for: Comparison of Drought and Heat Resistance Strategies among Six Populations of Solanum chilense and Two Cultivars of Solanum lycopersicum
Source: Plants (Basel). 2021 Aug 20;10(8):1720. doi: 10.3390/plants10081720 (PMC8398976; doi:10.3390/plants10081720)
Supplement: Supplementary file 1 [file plants-10-01720-s001.zip › plants-1326096-SI.pdf]

Table S1. Origin of the *S. chilense* populations

parameter	LA1930	LA2931	LA1958	LA2765	LA2880	LA4107
Province, Country	Arequipa, Peru	Tarapaca, Chile	Moquegua, Peru	Arica and Parinacota, Chile	Antofagasta, Chile	Antofagasta, Chile
Latitude Longitude	15° 17' 30'' S, 74° 36' 0'' W	20° 55' 0'' S, 69° 4' 0'' W	17° 15' 0'' S, 71° 15' 0'' W	18° 46' 0'' S, 69° 41' 0'' W	23° 49' 0'' S, 68° 13' 0'' W	25° 19' 8'' S, 70° 26' 46'' W
Altitude (m)	500	2275	1250	1400	2500	86
Temperature (°C)[a]	19.6	12.3	17.1	12.3	13.9	18.2
Precipitation (mm)[b]	6	18	6	51	36	22

Note: geographic data from the Tomato Genetics Resource Center, UC Davis (http://tgrc.usdavis.edu/), climatic data from WorldClim database (http://www.worldclim.org/, Böndel et al., 2014)

[a] mean annual temperature

[b] annual precipitation

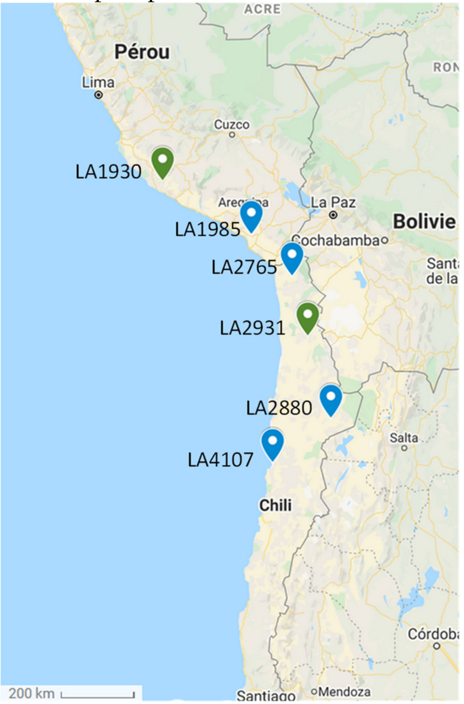

Geographical distribution of the *S. chilense* populations. Blue populations belong to the first group according to Principal component analysis (PCA) and green populations belong to the second group according to PCA (see Figure 1).

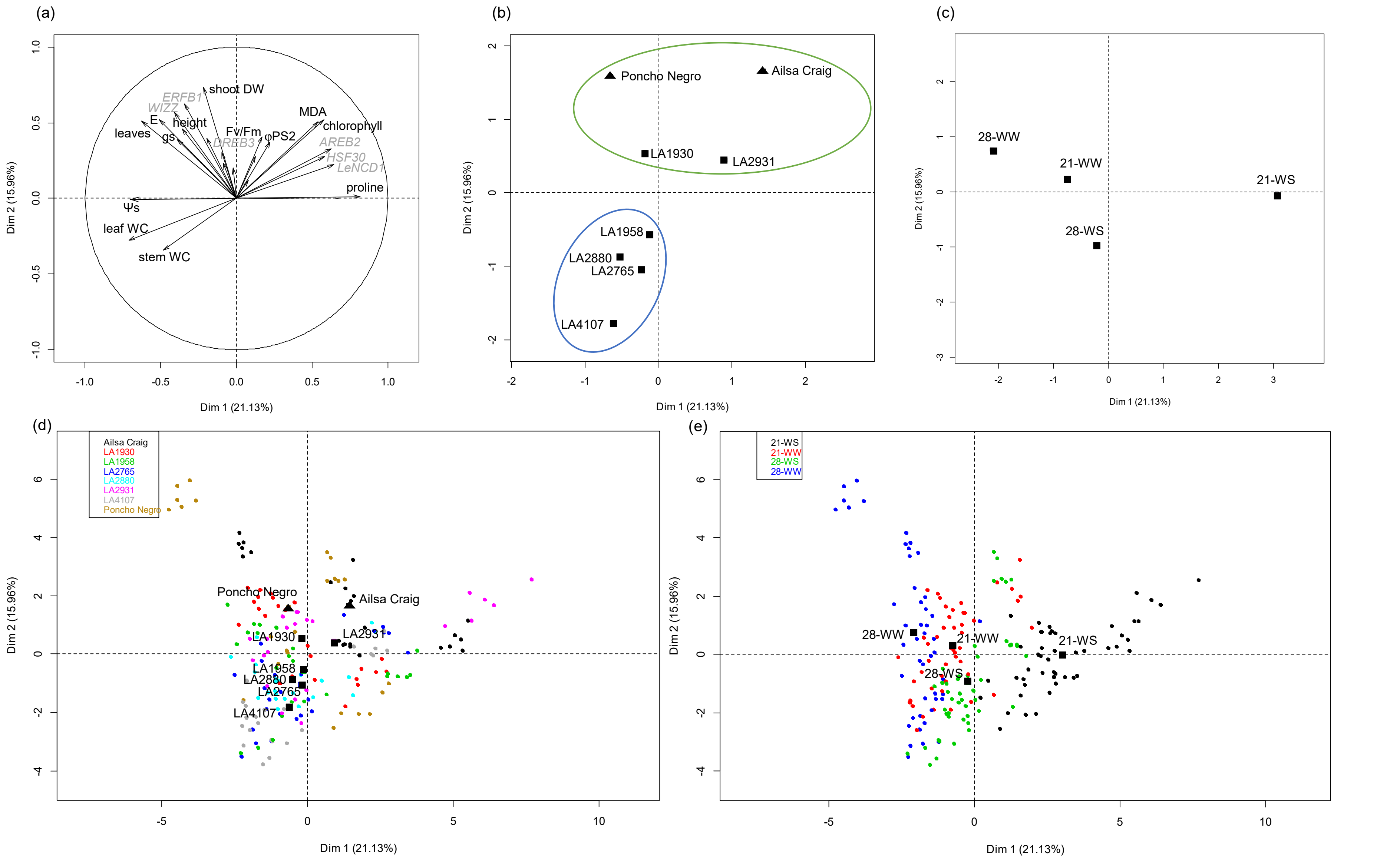

Figure S1. Principal component analysis (PCA) of growth parameters, physiological parameters and gene expression of stress markers in *S. lycopersicum* and *S. chilense* subjected to two temperatures (21°C vs. 28) and water supply (well-watered vs. water stress) conditions. (A) Variable graph of PCA showing growth and physiological parameters (black) and marker genes (grey); only significant parameters at P < 0.001 were shown. (B, D) Individual graph showing the accessions of *S. chilense* (square) and *S. lycopersicum* (triangle). (C, E) Individual graph showing the treatments. Only mean individuals are presented in (B, C) and all individuals are presents in (D, E). Dim 1 and Dim 2: dimension 1 and 2 of the PCA; DW : dry weight; gs: stomatal conductance; MDA: malondialdehyde; Ψs: osmotic potential; φPS2: efficiency of photosystem 2; WC: water content

Table S2. Plant growth and physiological parameters of *S. lycopersicum* (cv. Ailsa Craig and Poncho Negro) and *S. chilense* (populations LA1930, LA2931, LA1958, LA2765, LA2880, LA4107) grown under control conditions (21°C/19°C, 40% soil VWC) for 100 days.

**parameter**	**Ailsa Craig**	**Poncho Negro**	**LA1930**	**LA2931**	**LA1958**	**LA2765**	**LA2880**	**LA4107**	**ANOVA1**
**plant growth**									
Plant height (cm)	150 ± 7 a	145 ± 11 a	171 ± 9 a	136 ± 5 a	151 ± 8 a	135 ± 16 a	151 ± 17 a	79 ± 4 b	$F_{7,22}$ = 4.54, **P = 0.0029**
Number of leaves	23.3 ± 1.2 a	15.5 ± 1.5 ab	21.8 ± 3.0 ab	14.4 ± 1.5 ab	20.5 ± 1.5 ab	16.7 ± 1.2 ab	19.0 ± 3.0 ab	12.5 ± 0.5 b	$F_{7,22}$ = 8.56, **P = 0.0077**
Number of inflorescences	5.5 ± 0.4 a	2.5 ± 0.7 b	0.8 ± 0.4 b	1.4 ± 0.8 b	1.8 ± 0.9 b	2.5 ± 0.5 b	1.8 ± 0.8 b	2.7 ± 0.9 b	$F_{7,22}$ = 440, **P = 0.0015**
Number of ramifications	4.5 ± 0.4	3.2 ± 0.5	4.8 ± 2.8	8.2 ± 1.1	8.3 ± 3.0	8.0 ± 2.9	5.7 ± 1.8	9.7 ± 2.7	$F_{7,25}$ = 1.98, P = 0.0984
Shoot DW (g)	37.8 ± 2.8 ab	35.7 ± 1.4 ab	41.6 ± 2.7 a	34.3 ± 1.4 ab	21.7 ± 7.9 abc	18.1 ± 14.7bc	3.1 ± 1.1c	4.6 ± 1.8c	$F_{7,14}$ = 9.98, **P = 0.0003**
**Photosynthesis and water status**									
CCI	49.2 ± 2.3 a	40.7 ± 4.9 ab	43.3 ± 4.7 ab	37.8 ± 2.0 ab	38.2 ± 2.6 ab	32.0 ± 4.0 b	32.8 ± 4.0 ab	35.6 ± 1.0 ab	$F_{7,23}$ = 2.72, **P = 0.0330**
A (µmol CO_2 /m² s)	1.62 ± 0.46	1.53 ± 0.46	1.66 ± 0.72	2.20 ± 0.43	1.97 ± 0.27	2.14± 0.69	1.48 ± 0.67	2.02 ± 0.49	$F_{7,23}$ = 0.28, P = 0.9572
E (mmol H_2O/m²s)	3.37 ± 0.72	3.50 ± 0.20	2.75 ± 0.57	3.00 ± 0.14	2.38 ± 0.56	2.10 ± 0.54	3.61 ± 1.04	3.48 ± 0.34	$F_{7,23}$ = 1.09, P = 0.4042
Ci (µL/L)	394 ± 1.9	395 ± 4.7	393 ± 5.5	393 ± 2.9	385 ± 6.7	382 ± 5.6	388 ± 18	397 ± 2.3	$F_{7,23}$ = 0.69, P = 0.6815
gs (mol/m² s)	0.46 ± 0.17	0.39 ± 0.06	0.35 ± 0.15	0.32 ± 0.03	0.25 ± 0.09	0.18 ± 0.06	0.67 ± 0.29	0.44 ± 0.09	$F_{7,23}$ = 1.29, P = 0.2978
WUE (µmol CO_2/mmol H_2O)	0.45 ± 0.07	0.45 ± 0.15	0.62 ± 0.19	0.73 ± 0.13	0.94 ± 0.17	0.88 ± 0.25	0.72 ± 0.53	0.56 ± 0.11	$F_{7,23}$ = 0.78, P = 0.6093
Leaf WC (%)	90.3 ± 0.7 ab	89.9 ± 01.0 ab	88.9 ± 0.6 ab	89.1 ± 0.8 ab	90.7 ± 0.9 ab	90.0 ± 1.5 ab	87.1 ± 1.6 b	93.1 ± 0.9 a	$F_{7,14}$ = 3.13, **P = 0.0329**
Stem WC (%)	92.1 ± 0.3 a	91.9 ± 0.6 a	85.8 ± 1.1 b	86.6 ± 0.8 b	90.3 ± 1.3 a	91.6 ± 2.1 a	92.5 ± 2.3 a	93.4 ± 1.1 a	$F_{7,14}$ = 6.18, **P = 0.0019**
φPS2	0.78 ± 0.02	0.61 ± 0.14	0.81 ± 0.01	0.73 ± 0.04	0.75 ± 0.04	0.71 ± 0.09	0.57 ± 0.10	0.74 ± 0.06	$F_{7,23}$ = 1.18, P = 0.3498
NPQ	0.32 ± 0.09	0.30 ± 0.03	0.25 ± 0.05	0.27 ± 0.05	0.27 ± 0.04	0.24 ± 0.04	0.34 ± 0.02	0.27 ± 0.05	$F_{7,23}$ = 0.41, P = 0.8859
Ψs (MPa)	-0.81 ± 0.08	-0.83 ± 0.09	-0.76 ± 0.04	-0.66 ± 0.1	-0.71 ± 0.03	-0.78 ± 0.4	-0.66 ± 0.02	-0.61 ± 0.07	$F_{7,40}$ = 1.64, P = 0.1515
Proline (µmol/g FW)	0.12 ± 0.02 b	0.013 ± 0.006 c	0.43 ± 0.03 a	0.19 ± 0.01 b	0.12 ± 0.01 b	0.17 ± 0.004 b	0.14 ± 0.02 b	0.05 ± 0.008 c	$F_{7,16}$ = 64.58, **P < 0.0001**
MDA(nmol/g FW)	23.9 ± 1.9 a	21.7 ± 0.9 a	14.7 ± 0.3 bc	19.4 ± 2.9 ab	11.4 ± 0.4 c	13.4 ± 2.8 c	10.4 ± 0.6 c	8.4 ± 0.2 c	$F_{7,16}$ = 12.04, **P < 0.0001**

DW: dry weight, CCI: chlorophyll content index, A: photosynthesis rate, E: transpiration rate, Ci; intercellular CO_2 concentration, gs: stomatal conductance, WUE: water use efficiency, WC; water content, φPS2 : efficiency of photosystem 2, NPQ: non-photochemical quenching, Ψs: osmotic potential

For a given treatment, accessions followed by different letters are significantly different at $p < 0.05$ (only shown when significant difference for ANOVA1). Data ± SE. Different letters indicate statistically significant differences among groups at $p < 0.05$. If a letter is shared, the differences are not significant.

Table S3. Results of statistical analysis (ANOVA3) for the analyzed parameters

parameter	accession	temperature	water	accession x temperature	accession X water	Temperature x water	accession x temperature x water
**plant growth**							
Plant height (cm)[1]	F = 12.68, ***	F = 73.21, **	F = 0.23, P = 0.63	F = 0.71, P = 0.66	F = 0.81, P = 0.57	F = 5367, *	F = 9.38, **
# leaves[1]	F = 12.40, ***	F = 126.27, ***	F = 8.24, **	F = 1.40, P = 0.20	F = 4.30, ***	F = 1.21, P = 0.27	F = 0.26, P = 0.97
# inflorescences	F = 9.83, ***	F = 13.72, ***	F = 0.63, P = 0.42	F = 3.76, **	F = 8.50, ***	F = 0.01, P = 0.92	F = 4.75, ***
# ramifications	F = 17.29, ***	F = 17.69, ***	F = 11.60, **	F = 1.96, P = 0.06	F = 1.43, P = 0.19	F = 2.24, P = 0.14	F = 1.52, P = 0.17
Shoot DW (g)	F = 18.45, ***	F = 2.01, P = 0.16	F = 72.20, ***	F = 5.74, ***	F = 12.59, ***	F = 0.69, P = 0.41	F = 2.05, P = 0.06
**Photosynthesis and water status**							
CCI	F = 12.74, ***	F = 19.44, ***	F = 14.16, **	F = 1.75, P = 0.11	F = 1.00, P = 0.43	F = 3.97, *	F = 0.95, P = 0.47
A	F = 0.36, P = 0.92	F = 41.32, ***	F = 30.23, ***	F = 0.98 P = 0.45	F = 0.12, P = 0.99	F = 16.55, **	F = 0.45, P = 0.87
E	F = 5.15, ***	F = 4.19, *	F = 116.64, ***	F = 2.42, *	F = 3.26, **	F = 1.37, P = 0.24	F = 2.09, P = 0.053
Ci	F = 2.90, **	F = 0.66, P = 0.42	F = 1.48, P = 0.23	F = 0.94, P = 0.48	F = 1.45, P = 0.20	F = 5.30, *	F = 0.77, P = 0.61
gs	F = 5.35, ***	F = 12.70, **	F = 100.91, ***	F = 3.62, **	F = 3.16, **	F = 4.07, *	F = 2.58, *
WUE	F = 0.89, P = 0.52	F = 39.18, ***	F = 0.05, P = 0.82	F = 0.88, P = 0.52	F = 1.46, ¨= 0.19	F = 0.71, P = 0.40	F = 0.75, P = 0.63
NQP	F = 1.16, P = 0.34	F = 5.66, *	F = 2.86, P = 0.09	F = 1.59 P = 0.15	F = 1.41, P = 0.21	F = 6.13, *	F = 1.21, P = 0.31
φPS2	F = 2.97, **	F = 3.24, P = 0.07	F = 0.46, P = 0.49	F = 3.06, **	F = 0.43, P = 0.88	F = 0.28, P = 0.60	F = 0.41, P = 0.89
Leaf WC (%)	F = 3.24, **	F = 24.12, ***	F = 45.60, ***	F = 3.11, **	F = 1.76, P = 0.11	F = 14.04, ***	F = 2.38, *
Stem WC (%)	F = 3.68, **	F = 1.39, P = 0.24	F = 5.53, *	F = 1.51, P = 0.18	F = 0.37, P = 0.92	F = 6.03, *	F = 2.55, *
Ψs	F = 13.62, ***	F = 181.49, ***	F = 1177, ***	F = 10.11, ***	F = 4.18, **	F = 300.41, ***	F = 3.19, **
Proline	F = 123.19, ***	F = 2514, ***	F = 5193, ***	F = 44.39, ***	F = 89.96, ***	F = 1794, ***	F = 2802, ***
MDA	F = 70.59, ***	F = 73.90, ***	F = 76.07, ***	F = 6.70, ***	F = 19.29, ***	F = 14.49, ***	F = 11.99, ***
**Gene expression**							
AREB2	F = 20.11, ***	F = 231.26, ***	F = 1.32, P = 0.26	F = 18.75, ***	F = 6.03, ***	F = 3.92,P = 0.06	F = 16.15, ***
DREB3	F = 3.81 **	F = 8.52, **	F = 0.87, P = 0.35	F = 10.48 ***	F = 4.31, **	F = 31.18, ***	F = 4.05, **
ERFB1	F = 24.99, ***	F = 130.12, ***	F = 6.39, *	F = 39.45, ***	F = 3.97, **	F = 4.27, *	F = 3.69, **
HSF30	F = 69.46, ***	F = 62.05, ***	F = 39.10, ***	F = 25.69, ***	F = 8.08, ***	F = 0.92, P = 0.34	F = 4.51, **
LeNCED1	F = 20.86, ***	F = 114, ***	F = 14.40, ***	F = 7.18, ***	F = 1.75, P = 0.14	F = 7.27, **	F = 5.79, ***
NAC6	F = 15.67, ***	F = 12.54, ***	F = 0.63, P = 0.43	F = 20.75, ***	F = 4.59, **	F = 33.76, ***	F = 6.88, ***
pLC30-15	F = 11.38, ***	F = 1.27, P = 0.26	F = 1.69, P = 0.20	F = 12.11,***	F = 1.27, P = 0.29	F = 25.43, ***	F = 4.53, **
WIZZ	F = 14.79, ***	F = 49.64, ***	F = 23.15, ***	F = 15.09, ***	F = 6.87, ***	F = 53.02, ***	F = 8.19, ***

[1] statistical analysis for plant height and leaf number was based on the whole dataset and not only on the last date (plant number was mentioned as repeated effect).

Table S4. Heat and drought effect on the expression of abiotic stress responsive genes in *S. lycopersicum* cultivars and *S. chilense* populations.

accessions	treatment	*HSF30*	*LeNCED1*	*ERFB1*	*WIZZ*	*AREB2*	*NAC6*	*pLC30-15*	*DREB3*
Ailsa Craig	21-WW	6.24 ± 0.74 ab	3.39 ± 0.73 a	0.32 ± 0.09 b	0.19 ± 0.06 b	2.79 ± 0.25 a	0.95 ± 0.16 b	0.66 ± 0.13 a	0.29 ± 0.12 b
	21-WS	8.33 ± 2.07 a	2.69 ± 1.24 a	0.42 ± 0.26 b	0.41 ± 0.32 b	1.69 ± 0.21 b	0.40 ± 0.16 c	0.46 ± 0.12 a	0.61 ± 0.30 b
	28-WW	0.73 ± 0.1 6c	0.76 ± 0.12 b	4.47 ± 1.92 a	3.07 ± 1.29 a	0.71 ± 0.14 c	1.33 ± 0.26 a	0.97 ± 0.26 a	1.53 ± 0.52 a
	28-WS	4.28 ± 1.23 b	1.28 ± 0.20 b	2.28 ± 0.86 b	0.26 ± 0.20 b	0.91 ± 0.08 c	0.48 ± 0.10 c	0.85 ± 0.30 a	0.79 ± 0.29 b
Poncho Negro	21-WW	0.98 ± 0.16 b	2.68 ± 0.25 a	0.45 ± 0.15 b	0.32 ± 0.03 b	0.97 ± 0.09 a	0.58 ± 0.14 c	0.37 ± 0.14 b	0.54 ± 0.08 b
	21-WS	1.29 ± 0.33 b	2.96 ± 0.95a	0.66 ± 0.32 b	1.44 ± 0.14 b	0.81 ± 0.15 a	1.12 ± 0.14 b	0.58 ± 0.06 b	0.73 ± 0.27 b
	28-WW	0.54 ± 0.04 b	0.62 ± 0.13b	4.82 ± 0.46 a	13.41 ± 4.34 a	0.79 ± 0.06 a	1.60 ± 0.13 a	1.50 ± 0.11 a	2.15 ± 0.52 a
	28-WS	3.99 ± 1.56 a	2.24 ± 0.83a	5.54 ± 0.93 a	2.90 ± 0.85 b	1.04 ± 0.27 a	1.53 ± 0.20 a	1.62 ± 0.60 a	1.61 ± 0.15 a
LA2931	21-WW	1.61 ± 0.47 a	2.98 ± 0.14 b	1.25 ± 0.45 a	0.72 ± 0.09 b	1.18 ± 0.09 b	0.40 ± 0.04 b	1.41 ± 0.31 b	1.78 ± 0.43 a
	21-WS	2.29 ± 0.90 a	5.99 ± 2.55 a	0.49 ± 0.11 a	1.15 ± 0.27 b	2.14 ± 0.25 a	1.70 ± 0.56 b	2.35 ± 0.44 a	0.97 ± 0.57 a
	28-WW	0.46 ± 0.20 b	1.22 ± 0.30 b	1.02 ± 0.77 a	3.87 ± 2.01 a	1.03 ± 0.21 b	4.00 ± 1.32 a	1.43 ± 0.37 b	1.36 ± 0.71 a
	28-WS	0.35 ± 0.10b	0.92 ± 0.18 b	1.19 ± 0.24 a	1.74 ± 0.25 b	0.66 ± 0.03 c	2.01 ± 0.49 b	0.89 ± 0.34 b	0.92 ± 0.15 a
LA1958	21-WW	1.33 ± 0.10 b	3.20 ± 0.91 b	1.00 ± 0.28 b	0.46 ± 0.12 b	1.55 ± 0.16 a	0.86 ± 0.04 a	0.65 ± 0.12 b	0.55 ± 0.13 b
	21-WS	2.17 ± 0.64 a	6.03 ± 0.8 a	0.28 ± 0.11 c	1.50 ± 0.39 b	1.73 ± 0.32 a	1.46 ± 0.58 a	1.44 ± 0.72 ab	1.03 ± 0.23 b
	28-WW	0.43 ± 0.12 c	1.90 ± 0.11 bc	2.41 ± 0.04 a	6.55 ± 3.38 a	0.90 ± 0.14 b	1.45 ± 0.57 a	1.95 ± 0.22 a	2.56 ± 0.61 a
	28-WS	0.66 ± 0.06 c	0.93 ± 0.08 c	1.00 ± 0.11 b	0.91 ± 0.20 b	0.61 ± 0.16 b	1.64 ± 0.33 a	0.88 ± 0.27 b	0.99 ± 0.07 b
LA2765	21-WW	0.83 ± 0.19 b	1.67 ± 0.09 b	0.85 ± 0.25 b	0.74 ± 0.09 ab	1.11 ± 0.39 b	0.77 ± 0.08 b	0.61 ± 0.12 b	0.86 ± 0.22 b
	21-WS	1.38 ± 0.49 a	3.51 ± 1.62 a	1.62 ± 0.27 a	1.65 ± 0.83 a	1.57 ± 0.07 a	1.40 ± 0.36 a	1.12 ± 0.13 a	2.70 ± 1.12 a
	28-WW	0.59 ± 0.11 b	0.51 ± 0.04 b	1.26 ± 0.16 a	0.20 ± 0.03 b	0.52 ± 0.13 c	0.69 ± 0.18 b	0.73 ± 0.09 ab	1.40 ± 0.35 b
	28-WS	0.27 ± 0.08 b	0.86 ± 0.14 b	0.65 ± 0.08 b	0.90 ± 0.06 ab	0.59 ± 0.05 c	0.99 ± 0.09	1.01 ± 0.33 ab	0.92 ± 0.22 b
LA4107	21-WW	0.44 ± 0.14 b	0.08 ± 0.01 a	0.74 ± 0.13 a	0.66 ± 0.17 b	0.78 ± 0.11 b	0.99 ± 0.09 b	1.39 ± 0.31 ab	0.80 ± 0.04 a
	21-WS	1.01 ± 0.17 a	0.11 ± 0.08 a	0.80 ± 0.33 a	1.36 ± 0.43 a	1.31 ± 0.42 a	2.09 ± 0.35 a	2.13 ± 0.57 a	1.55 ± 0.66 a
	28-WW	0.30 ± 0.09 b	0.04 ± 0.02 a	0.91 ± 0.01 a	1.53 ± 0.28 a	0.74 ± 0.18 b	0.60 ± 0.02 c	1.41 ± 0.40 ab	0.87 ± 0.17 a
	28-WS	0.36 ± 0.11 b	0.04 ± 0.01 a	0.73 ± 0.19 a	0.37 ± 0.09 b	0.64 ± 0.10 b	0.27 ± 0.12 c	0.98 ± 0.36 b	0.77 ± 0.07 a

Means and SD of expression level relative to the reference genes (*PP2Acs: Solyc05g006590, CAC: Solyc08g006960* and *TIP41: Solyc10g049850). S. lycopersicum* cv. Ailsa Craig (AC) and Poncho Negro (PN); *S. chilense* populations LA2931 LA1958, LA2765, LA4107. *HSF30* : Solyc08g062960, *LeNCED1* : Solyc07g056570, *ERFB1* : Solyc05g052040, *WIZZ* : Solyc03g116890, *AREB2* : Solyc11g044560, *NAC6* : Solyc10g055760, PLC30 : Solyc04g082200, *DREB3* : Solyc04g072900.

Treatments followed by different letters are significantly different at $p < 0.05$ for a same accession and gene. Different letters indicate statistically significant differences among groups at $p < 0.05$. If a letter is shared, the differences are not significant.

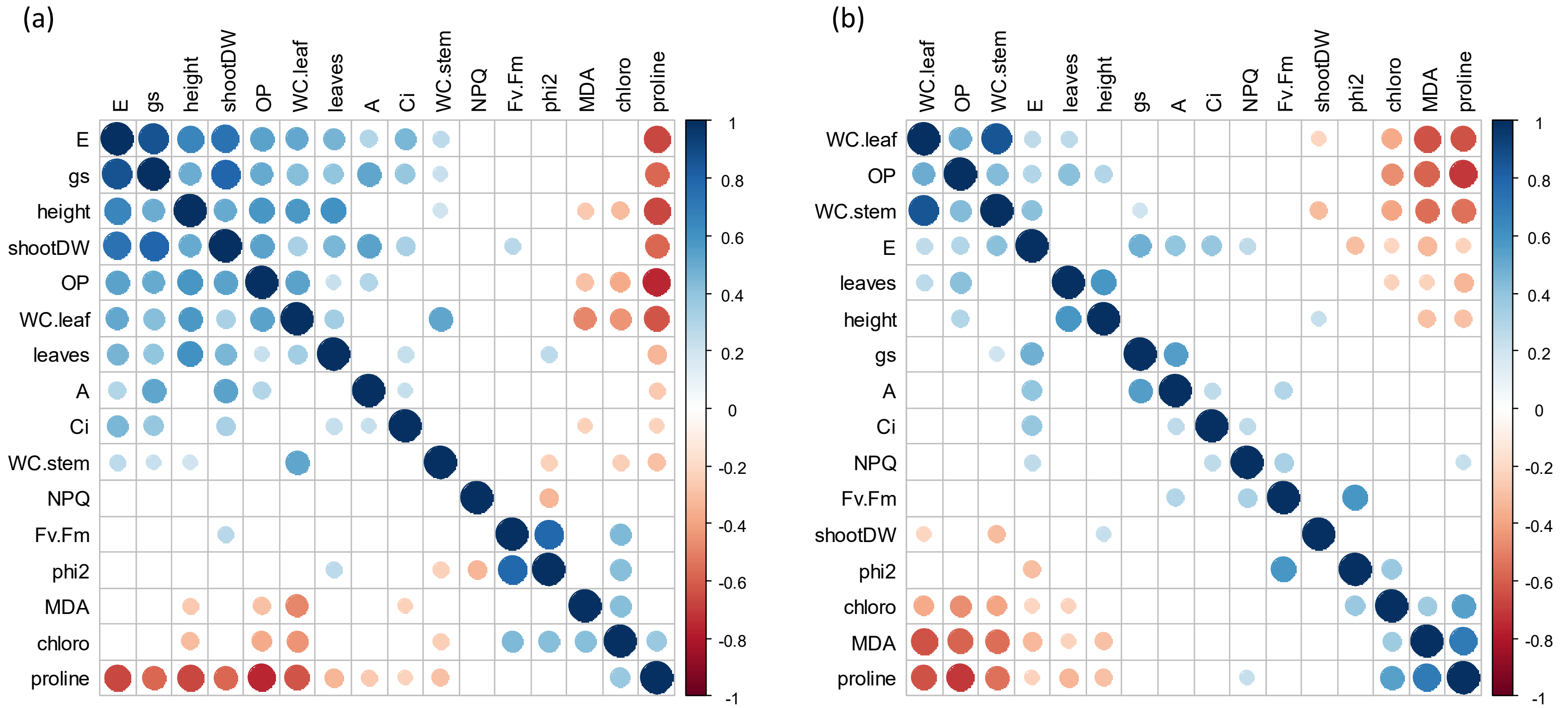

Figure S2. Correlation plots between plant growth and physiological parameters in (a) sensitive accessions and (b) resistant accessions. Only significant Pearson correlations at 5% were shown. DW: dry weight, CCI: chlorophyll content index, A: photosynthesis rate, E: transpiration rate, Ci; intercellular CO_2 concentration, gs: stomatal conductance, WUE: water use efficiency, WC; water content, phi2 : efficiency of photosystem 2, NPQ: non-photochemical quenching, OP: osmotic potential, chloro: chlorophyll concentration, MDA: malondialdehyde.

Table S5. List of genes and primers used in this study.

Name gene	Accession Number	primers (primer sequence 5'-3')	Amplicon length (bp)	Gene description	reference
Abiotic stress-responsive genes					
AREB2	Solyc11g044560	F: TGCAGAGACAGGGTTCTTTG<br>R: CTGCCCAAAGTTTGATCCTC	134	AREB/ABF subfamily of basic leucine zipper (bZIP) transcription factor	[1]
DREB3	Solyc04g072900	F: TTACAGACTGCCCATCATGC<br>R: TAGAGCTTCGTGGGTTTTGG	98	AP2/EREP transcription factor family	[1]
ERFB1	Solyc05g052040	F: GAATGATGACGGAATTGTAATGAAGA<br>R: TTCCACAATCCCAAATTGAAGA	101	Ethylene responsive transcription factor 1	[2]
HSF30	Solyc08g062960	F: TATGGAACCAGTGGCTGATG<br>R: CCCCATTCAGGTGTTTTCAC	150	Heat stress transcription factor A3	[3]
LeNCED1	Solyc07g056570	F: CGGCGATCTTAAAACAGAGG<br>R: TTGGGTGAGCTATCATGGTG	71	9-cis-epoxycarotenoid dioxygenase	[4]
NAC6	Solyc10g055760	F: CGTCAATCAGCAAGGATGAAG<br>R: TTGGGTACTTGCAGTTCAGC	145	NAC domain protein NAC6	[3]
pLC30-15	Solyc04g082200	F: ACGTGGAAGCTACTGATCGTG<br>R: TCCTTGGGTTCCACTTCTTC	142	group 2 LEA (dehydrin)	[1]
WIZZ	Solyc03g116890	F: CGGTAATGCCAAGACAAACC<br>R: TTACGCTTCTGACCACTTGC	118	WRKY transcription factor 2	[3]
Reference genes					
PP2Acs	Solyc05g006590	F: GAAACCATGAAAGCCGACAG<br>R: TCTATGAGTGCCGTCAATGG	133	protein phosphatase 2A catalytic subunit 1	[5]
CAC	Solyc08g006960	F:TCTCTACAGGGACGATGTTGG<br>R: AAGAACAGCCTCCAATCTGC	110	AP-2 complex subunit mu	[5]
TIP41	Solyc10g049850	F: CGAAAGCAATGTCGAGTGTG<br>R: GGTTCTTTAGACGCCAATGC	111	TIP41-like protein	[6]
TUB	Solyc04g081490	F: AACCTCCATTCAGGAGATGTT<br>R: TCTGCTGTAGCATCCTGGTATT	180	Tubulin	[5]
UBI	Solyc07g064130	F: GGACGGACGTACTCTAGCTGAT<br>R: AGCTTTCGACCTCAAGGGTA	134	Ubiquitin	[5]

1. Böndel, K.B.; Nosenko, T.; Wolfgang, S. Signatures of natural selection in abiotic stress-responsive genes of Solanum chilense. *Royal Society Open Science* **2018**, *5*, 171198, doi:10.1098/rsos.171198.
2. Klay, I.; Gouia, S.; Liu, M.; Mila, I.; Khoudi, H.; Bernadac, A.; Bouzayen, M.; Pirrello, J. Ethylene Response Factors (ERF) are differentially regulated by different abiotic stress types in tomato plants. *Plant Sci.* **2018**, *274*, 137–145, doi:10.1016/j.plantsci.2018.05.023.
3. Gong, P.; Zhang, J.; Li, H.; Yang, C.; Zhang, C.; Zhang, X.; Khurram, Z.; Zhang, Y.; Wang, T.; Fei, Z.; et al. Transcriptional profiles of drought-responsive genes in modulating transcription signal transduction, and biochemical pathways in tomato. *J. Exp. Bot.* **2010**, *61*, 3563–3575, doi:10.1093/jxb/erq167.

4. Xia, H.; Camus-Kulandaivelu, L.; Stephan, W.; Tellier, A.; Zhang, Z. Nucleotide diversity patterns of local adaptation at drought-related candidate genes in wild tomatoes. *Molecular Ecology* **2010**, *19*, 4144–4154, doi:https://doi.org/10.1111/j.1365-294X.2010.04762.x.
5. Løvdal, T.; Lillo, C. Reference Gene Selection for Quantitative Real-Time PCR Normalization in Tomato Subjected to Nitrogen, Cold, and Light Stress. Anal Biochem 2009, 387, 238–242, doi:10.1016/j.ab.2009.01.024.
6. Leelatanawit, R.; Saetung, T.; Phuengwas, S.; Karoonuthaisiri, N.; Devahastin, S. Selection of Reference Genes for Quantitative Real-Time PCR in Postharvest Tomatoes (*Lycopersicon esculentum*) Treated by Continuous Low-Voltage Direct Current Electricity to Increase Secondary Metabolites. International Journal of Food Science & Technology 2017, 52, 1942–1950, doi:10.1111/ijfs.13477.
